# *Burkholderiaceae* Are Key Acetate Assimilators During Complete Denitrification in Acidic Cryoturbated Peat Circles of the Arctic Tundra

**DOI:** 10.3389/fmicb.2021.628269

**Published:** 2021-02-05

**Authors:** Stefanie A. Hetz, Marcus A. Horn

**Affiliations:** Institute of Microbiology, Leibniz University Hannover, Hannover, Germany

**Keywords:** 16S rRNA stable isotope probing, nitrous oxide, climatechange, permafrost affected soils, isotope tracing

## Abstract

Cryoturbated peat circles (pH 4) in the Eastern European Tundra harbor up to 2 mM pore water nitrate and emit the greenhouse gas N_2_O like heavily fertilized agricultural soils in temperate regions. The main process yielding N_2_O under oxygen limited conditions is denitrification, which is the sequential reduction of nitrate/nitrite to N_2_O and/or N_2_. N_2_O reduction to N_2_ is impaired by pH < 6 in classical model denitrifiers and many environments. Key microbes of peat circles are important but largely unknown catalysts for *C*- and *N*-cycling associated N_2_O fluxes. Thus, we hypothesized that the peat circle community includes hitherto unknown taxa and is essentially unable to efficiently perform complete denitrification, i.e., reduce N_2_O, due to a low *in situ* pH. 16S rRNA analysis indicated a diverse active community primarily composed of the bacterial class-level taxa Alphaproteobacteria, Acidimicrobiia, Acidobacteria, Verrucomicrobiae, and Bacteroidia, as well as archaeal Nitrososphaeria. Euryarchaeota were not detected. ^13^C_2_- and ^12^C_2_-acetate supplemented anoxic microcosms with endogenous nitrate and acetylene at an *in situ* near pH of 4 were used to assess acetate dependent carbon flow, denitrification and N_2_O production. Initial nitrate and acetate were consumed within 6 and 11 days, respectively, and primarily converted to CO_2_ and N_2_, suggesting complete acetate fueled denitrification at acidic pH. Stable isotope probing coupled to 16S rRNA analysis via Illumina MiSeq amplicon sequencing identified acetate consuming key players of the family *Burkholderiaceae* during complete denitrification correlating with *Rhodanobacter* spp. The archaeal community consisted primarily of ammonia-oxidizing Archaea of Nitrososphaeraceae, and was stable during the incubation. The collective data indicate that peat circles (i) host acid-tolerant denitrifiers capable of complete denitrification at pH 4–5.5, (ii) other parameters like carbon availability rather than pH are possible reasons for high N_2_O emissions *in situ*, and (iii) *Burkholderiaceae* are responsive key acetate assimilators co-occurring with *Rhodanobacter* sp. during denitrification, suggesting both organisms being associated with acid-tolerant denitrification in peat circles.

## Introduction

Nitrous oxide (N_2_O) is a potent greenhouse gas with a global warming potential about 300 times higher than CO_2_, and a long atmospheric half-life of estimated 120 years (Prather et al., 2015; Stocker et al., 2018). The main source of N_2_O is microbial denitrification, i.e., the sequential reduction of nitrate (NO_3_^–^) or nitrite (NO_2_^–^) via the intermediates nitric oxide (NO) and N_2_O to dinitrogen gas (N_2_) under the exclusion of oxygen (Zumft, 1997). Different reductases are involved in this process. The first step in the process is facilitated via the dissimilatory nitrate reductase Nar, a membrane bound enzyme (Zumft, 1997). The reduction of NO_2_^–^ to NO can be executed by either the cytochrome *cd*_1_ dependent nitrite reductase cd-Nir or one of three known types of copper-dependent nitrite reductases CuNir (Zumft, 1997; Helen et al., 2016). The cytotoxic gas NO can then be further reduced to N_2_O via the NO reductases cNOR, associated with cytochrome *c*, the copper-dependent Cu-qNor or the quinol dependent qNOR (Zumft, 2005). The last step of denitrification is catalyzed by the copper-containing N_2_O-reductases NosZ, the only known enzyme capable of this reaction (Zumft, 1997; Jones et al., 2008). More than 60 genera, within archaea, bacteria, and fungi, are known to be capable of denitrification, displaying a broad phylogenetic and functional variability (Zumft, 1997; Philippot et al., 2007). Since many organisms only possess the genetic potential to perform parts of the whole denitrification process, truncated forms lacking N_2_O reductases exist, which can lead to the release of N_2_O as end product, contributing to N_2_O emissions from soils (Cofman Anderson and Levine, 1986; Stein and Klotz, 2016).

Many studies focus on denitrification and associated fluxes N_2_O in diverse environments including permafrost affected soils and sediments (Braker and Conrad, 2011; Albina et al., 2019; Kumar et al., 2020; Hetz and Horn, 2021). Tropical rainforest soils have the highest known N_2_O emission potentials (Potter et al., 1996; Werner et al., 2007). These soils offer ideal conditions for denitrification, with a high supply of mineral nitrogen and an optimum soil moisture (Breuer et al., 2000). In contrast, permafrost affected soils are traditionally viewed as sources of the greenhouse gas methane rather than N_2_O, nitrogen limited, slowly mineralizing organic matter, and not contributing significantly to the global N_2_O budget (Nadelhoffer et al., 1991; Shaver et al., 1992; Jonasson et al., 1999). Permafrost soils cover approximately 17% of Earth’s surface, and only a decade ago cryoturbated peat circles were found to emit N_2_O in the range of temperate agricultural and (sub)tropical rainforest soils during growing season (1.9–31 mg N_2_O m^–2^ d^–1^; Repo et al., 2009; Marushchak et al., 2011; Gruber, 2012), emphasizing the emerging awareness of permafrost regions for N-cycle derived greenhouse gas emissions (reviewed in Hetz and Horn, 2021). Such peat circles are thus significant sources of N_2_O accounting for up to 0.6% of annual global N_2_O emissions (Christensen, 1993; Denman et al., 2007; Repo et al., 2009). A low C/N ratio of old peat material, an oxic/anoxic interface, the lack of vegetation as competitor for nitrogen, high nitrification activities by archaeal ammonia-oxidizers, and intermediate water content, account for high NO_3_^–^ concentrations of up to 2 mM in the pore water of peat circles, which is one of the main sources of N_2_O in soils and readily available for denitrifiers (Repo et al., 2009; Siljanen et al., 2019). A major parameter determining the emission ratio of N_2_O/N_2_ from soils is pH, leading to an increased release of N_2_O relative to N_2_ at low pH due to an impaired N_2_O reduction (Simek and Cooper, 2002; Cuhel et al., 2010; Bergaust et al., 2012), suggesting that a low pH is a major reason for high N_2_O emissions of peat circles. Indeed, only very few strains, all belonging to the genus *Rhodanobacter*, have been associated with N_2_O reduction at low pH to date (Van Den Heuvel et al., 2010; Prakash et al., 2012; Lycus et al., 2017).

Denitrifier community analysis revealed that peat circle denitrifiers are only distantly related to known denitrifiers (Palmer et al., 2012). Functional gene analysis identified the genetic potentials for complete denitrification to N_2_. Phylogenetic affiliations of *nosZ* genes showed a high relative abundance of Alphaproteobacterial *nosZ* (*Mesorhizobium* sp.), of which 60 % were only distantly related to *nosZ* of cultured microorganisms, indicating a new, specific, and acid-tolerant denitrifier community with little N_2_O reduction capacity in these soils (Palmer et al., 2012). In contrast, unturbated vegetated peat soils from the same study site with the same acidic pH, do not emit N_2_O *in situ* (Repo et al., 2009; Marushchak et al., 2011). Phylogenetic functional gene data show that denitrifier communities differ between bare cryoturbated and vegetated unturbated peat soils, and are likely accountable for contrasting N_2_O emissions between soils rather than soil pH alone (Repo et al., 2009; Marushchak et al., 2011; Palmer et al., 2012). However, functional gene based phylogeny might be biased due to horizontal gene transfer and gene duplication events. 16S rRNA genes as phylogenetic markers are thus preferable for the analysis of community structure and to verify phylogenetic novelty. Interactions of microbes potentially impacting N_2_O fluxes, e.g., via competition for electron donors and carbon sources demand the analysis of the whole microbial community rather than denitrifiers alone.

Denitrifiers use low molecular weight organic carbon (LMWOC) as carbon source and electron donors in many peatland systems and sediments (Castaldelli et al., 2013; Boylan et al., 2020). LMWOC represents common intermediates in the anerobic food chain (McInerney and Bryant, 1981; Wüst et al., 2009). The anaerobic food chain is also referred to as intermediary ecosystem metabolism to highlight the complex network of trophically interacting physiological groups of microorganisms finally leading to methane in the absence of alternative electron acceptors other than CO_2_ (Wüst et al., 2009). The intermediary ecosystem metabolism includes hydrolysis of biopolymers to monomers, primary and secondary fermentations, acetogenesis, and finally methanogenesis. Acetate is one of the most often detected intermediates in peatlands and a prominent methane precursor (Zeikus et al., 1975). Methanogenesis and most of the other reactions are catalyzed by methanogenic Euryarchaeota and Bacteria, respectively. When alternative electron acceptors like nitrate are present, intermediary electron and carbon flow is diverged from methanogenesis to nitrate reduction and/or denitrification as terminal electron accepting processes (Tiedje, 1988). Indeed, high denitrification potentials were identified in cryoturbated peat circles in the presence of nitrate under anoxic conditions, and acetate was shown to stimulate denitrification (Palmer et al., 2012). Although acetate is undoubtedly an important intermediate in intermediary ecosystem metabolism, acetate derived carbon and electron flow and divergence to CO_2_ and/or nitrate in cryoturbated peat circles are unclear to date. Key players catalyzing such reactions in peat circle sediments are likewise unknown. Thus, we hypothesize that the peat circle community couples acetate consumption to incomplete denitrification yielding primarily N_2_O as end product due to a low *in situ* pH. Therefore, the main objectives of this study were 1) to determine the diversity of peat circle Bacteria and Archaea by 16S rRNA analysis, 2) analyze acetate derived carbon- and nitrate derived nitrogen-flow in anoxic microcosms, and 3) determine key acetate assimilators during active denitrification by rRNA dependent stable isotope probing. Many acetate assimilating microbes are chemoorganoheterotrophs that utilize acetate as carbon source and electron donor, i.e., dissimilate acetate to conserve energy in the form of a proton motive force and ATP by oxidizing acetate to CO_2_. It is general knowledge that acetate assimilation and dissimilation proceed via central metabolic pathways like the citric acid cycle, thus allowing for an easy partitioning of acetate carbon to oxidation to CO_2_ and assimilation. Our experimental approach included ^13^C-tracing, i.e., determining oxidation of ^13^C-labeled acetate carbon to ^13^CO_2_ during denitrification in peat circle sediments, calculation of assimilated acetate carbon, and sequence analysis of 16S rRNA of microbes that assimilated ^13^C-acetate carbon.

## Materials and Methods

### Site Description and Sampling

The study site was located in the Northeastern European Tundra in Russia within the discontinuous permafrost zone (67°03′N, 62°57′E, 100 m a.s.l.) with a mean annual air temperature of −5.6°C (Marushchak et al., 2011). Samples were taken from cryoturbated peat circles, which were previously described (Repo et al., 2009; Hugelius et al., 2011; Biasi et al., 2014). Generally, the carbon to nitrogen (C/N) ratio is low (23 ± 2) in cryoturbated peat circles and during growing season high amounts of N_2_O are emitted (1.9–31 mg N_2_O m^–2^ d^–1^) (Repo et al., 2009). Thawed sediment from the upper 10 cm of three peat circles was sampled from the active layer in summer 2014 (total thaw depth approximately 60 cm; 13.0 ± 0.3 °C mean temperature in 2 cm of depth; Marushchak et al., 2011), placed in gas-tight ZipLoc bags, and stored at 4°C until further processing to minimize potential changes in the microbial community during transport and storage. Experiments were conducted within three months after sampling. Soil moisture content was determined via weighing soil samples before and after drying at 60°C for 1 week, and was 74 ± 8.6% (*n* = 3).

### Preparation of Microcosms, Incubation and Sampling

Sediment of three sampled peat circles was pooled to provide a representative sample for peat circles with low variability, homogenized, and larger debris was removed prior to incubation. Soil slurries with an *in situ* near pH of 4.4 were prepared by mixing soil with deionized water (1:12) to a final volume of 300 ml in 500 ml veral bottles. The bottles were sealed with an airtight rubber stopper. Microcosms were prepared in triplicates for each treatment and incubated in the dark at 15°C, which is representative of the *in situ* peat circle temperature in 2 cm of depth during the growing season of 13.0 ± 0.3°C (Marushchak et al., 2011). Microcosms were rigorously shaken twice a day to minimize the formation of micro-gradients. The gas phase consisted of 100% nitrogen. In order to differentiate between complete and incomplete denitrification to N_2_O, microcosms with and without acetylene (10% vol/vol headspace) were prepared. Acetylene blocks the N_2_O reductase, hence N_2_O cannot be further reduced to N_2_ (Yoshinari et al., 1977). All microcosms contained the peat circle sediment endogenous nitrate (app. 300 μM). A nitrate depletion step and thus controls without nitrate were omitted due to modification of the microbial community and little *in situ* relevance. In total, 12 anoxic microcosms were prepared [2 (+/− acetylene) × 2 ([^13^C_2_]- or ([^12^C]- acetate) × 3 (triplicates); Supplementary Figure S1]. Microcosms were supplemented with 600 μM nitrate as soon as endogenous nitrate was depleted. Isotope labeling of microorganisms was initiated by supplementing ^13^C-labeled [^13^C_2_]-acetate (99 atom-%, Sigma-Aldrich, MO, United States) to a final concentration of 400 μM, which was refed three times to maximize ^13^C-labeling of acetate assimilators. Control microcosms received unlabeled acetate. N_2_O, nitrate, and acetate were determined at regular intervals (Palmer et al., 2010). For the microbial community analyses, microcosms were sampled at the start and the end of incubation (16 day time span). A total of 20 ml slurry sample were immediately suspended in 2.5 ml RNA stabilization buffer (100 mM sodium acetate, 100 mM EDTA, pH 5.2) together with 1 ml 20% SDS, 64 μl mercaptoethanol, and 2 ml equilibrated phenol. In order to avoid decomposition of nucleic acids, samples were flash-frozen in liquid nitrogen and stored at −80°C until further processing.

### Analytical Methods and Statistics

Gases (N_2_O, CH_4_, and CO_2_) were measured via gaschromatography coupled to electron capture, flame ionization, andthermal conductivity detection, respectively (Horn et al., 2003; Palmer et al., 2010; Hunger et al., 2011). [^13^C/^12^C]-isotope ratios of CO_2_ were determined by GC combustion-isotope ratio mass spectrometry (GC-C-IRMS; BayCEER – Laboratories for Isotopic-Biogeochemistry, University of Bayreuth, GER). Liquid samples were analyzed for soluble organic compounds via high performance liquid chromatography (Palmer et al., 2010). Determination of ^13^C labeled soluble compounds was done via HPLC-ESI-MS (BayCEER – Atmospheric Chemistry, University of Bayreuth, GER). Sulfate, nitrate, nitrite, ammonium, and iron(II) were measured by colorimetric assays (Harrigan and McCance, 1966; Cataldo et al., 1975; Gadkari, 1984; Wüst et al., 2009). Statistical analyses were performed in OriginPro 2020 (OriginalLab Corporation, Northampton, MA, United States). Prior to statistical tests, basic data analyses were performed, including visual inspection of all measured variables coupled with the Shapiro–Wilk normality test. Analysis of variance (ANOVA) was used to test for the treatment effect, i.e., differences between controls and supplemented microcosms.

### RNA Extraction and Density Gradient Fractionation

Prior to nucleic acid extraction, it was assured that all solutions and glassware were RNase free by either treatment with DEPC or heat sterilization (180°C, 8 h), respectively. Utilized plasticware was certified DNase- and RNase-free. Nucleic acids were extracted in triplicates, samples analyzed included *t*_0_ (before incubation) and *t*_*end*_ (after incubation) of treatments incubated without acetylene. The coextraction of DNA and RNA followed a modified protocol of Griffiths et al. (2000). Prior to extraction, a wash step modified after Placella et al. (2012) was implemented to remove humic substances that were highly present in the samples. Pure RNA was retrieved by treating pooled nucleic acid extracts with DNAse I (RNase free, New England Biolabs, MA, United States). Digestion success was verified via 16S rRNA gene amplification and visualization on agarose gel. RNA was quantified with RiboGreen (Thermo Fisher Scientific, MA, United States), and 500 ng of RNA per sample were loaded onto a CsTFA gradient buffer (Whiteley et al., 2007). After isopycnic density gradient centrifugation for 67 h at 20°C at 130,000 *g*_*av*_, 10 fractions of each sample were collected, and RNA precipitated for subsequent community analyses (Dallinger and Horn, 2014). Buoyant density (BD) of each fraction was determined by weighing of fractions obtained from a blank gradient. Heavy and light fractions were defined according to values from literature, with heavy fractions ranging between 1.818 and 1.824 g ml^–1^ and light fractions ranging between 1.770 and 1.784 g ml^–1^ (Lueders et al., 2003).

### Denaturing Gradient Gel Electrophoresis (DGGE) Analysis of Density-Resolved rRNA

RNA fractions were pairwise pooled resulting in five pooled fractions (1–2, 3–4, 5–6, 7–8, and 9–10) per gradient and subjected to reverse transcription (SuperScript IV, Thermo Fisher Scientific, MA, United States) according to the manufacturer’s protocol. Pooled fractions recovered from CsTFA gradients were compared by denaturing gradient gel electrophoresis (DGGE) fingerprinting to determine the success of labeling prior to amplicon sequencing. Primers Bact340F (TAC GGG AGG CAG CAG; Li et al., 2010) and 907R (CCG TCA ATT CMT TTG AGT TT; Muyzer et al., 1995) were used to amplify the 16S rRNA gene for DGGE, with the forward primer containing a G + C rich sequence at the 5′ end (CGC CCG CCG CGC CCC GCG CCC GTC CCG CCG CCC CCG CCC GCC; clamp; Muyzer et al., 1993). PCR reactions were carried out as 40 μl reactions, containing 1× SensiMix SYBR & Fluorescein (Bioline, London, United Kingdom), 500 nM of each primer and 4 μl of template cDNA. Initial denaturation was performed at 94°C for 8 min. Denaturation, annealing and elongation were at 94°C for 30 s, 55°C for 30 s and 72°C for 60 s, respectively, with a total of 35 cycle, followed by a final elongation at 72°C for 5 min. Amplification was checked on a 1% agarose gel. Amplicons were then resolved on a 35 – 65% DGGE gradient gel (63 V, 60°C, 16.5 h) and imaged after the run was complete (Supplementary Figure S5) (Horn et al., 2003).

### 16S rRNA Gene Amplification and Amplicon Sequencing

Paired end Illumina MiSeq amplicon sequencing of the archaeal and bacterial 16S rRNA gene of representative pooled fractions was performed. Amplicon libraries for Archaea and Bacteria were generated with primer pairs A519F (CAG CMG CCG CGG TAA; Wang and Qian, 2009)/Arch1017R (GGC CAT GCA CCW CCT CTC; Yoshida et al., 2005) and 341F (CCT ACG GGN GGC WGC AG; Herlemann et al., 2011)/805R (GAC TAC HVG GGT ATC TAA TCC; Herlemann et al., 2011), respectively. Both forward and reverse primers were fused to adapter sequences at their 5′ end (TCG TCG GCA GCG TCA GAT GTG TAT AAG AGA CAG and GTC TCG TGG GCT CGG AGA TGT GTA TAA GAG ACA G, respectively). For each PCR, 40 μl reactions were set up, containing 1× SensiMix SYBR & Fluorescein (Bioline, London, United Kingdom), 500 nM of each primer and 4 μl of template cDNA. Initial denaturation was performed at 94°C for 8 min. Denaturation, annealing and elongation were at 94°C for 40 s, 53°C for 40 s and 72°C for 50 s for amplification of bacterial 16S rRNA derived cDNA, with a total of 30 cycles. For amplification of archaeal 16S rRNA derived cDNA, denaturation, annealing, and elongation were at 94°C for 30 s, 55.5°C for 30 s and 72°C for 30 s, with a total of 30 cycles. Terminal elongation was at 72°C for 5 min, for both protocols. PCR products were checked for right amplicon size on a 1% agarose gel and then purified with the GeneRead Size Selection Kit (Qiagen, Hilden, GER), before sequencing on the Illumina MiSeq platform (v3 chemistry) at the University of Göttingen (Genomic and Applied Microbiology, University of Göttingen, Germany).

### Sequence Processing

Sequence analysis was performed using mothur v.1.39.5 (Schloss et al., 2009) and a modified standard operational protocol for MiSeq data (Kozich et al., 2013). After paired-end joining of sequence reads, sequences were filtered by amplicon length, and sequences with ambiguous bases as well as duplicate sequences were removed. Next, sequences were aligned according to a reference database (Silva database^1^ v138; Quast et al., 2012; Yilmaz et al., 2014), formatted to be compatible with mothur^2^ (2019 Patrick D. Schloss, PhD), with maximum homopolymer length ≤8. Sequences were pre-clustered, allowing one difference for every 100 bp of sequence, and chimeras were identified and removed using VSEARCH (v2.6.0; Rognes et al., 2016). After classification against the Silva database (Silva database v138; Quast et al., 2012; Yilmaz et al., 2014) non-target sequences (e.g., fragments of mitochondria, eukaryota) were removed. Operational taxonomic units (OTUs) were assigned at 97% similarity level using the OptiClust algorithm (OTU assembly using metrics to determine the quality of clustering). Coverage and α-diversity indices were calculated using Good’s coverage (Good, 1953) and the Inverse Simpson diversity index (Simpson, 1949), respectively, and Bray–Curtis dissimilarity matrices were used for comparison of β-diversity (Bray and Curtis, 1957). Quantification and statistical inference of systematic changes between conditions were tested with the differential analysis of count data (DESeq2 package; Love et al., 2014). All other analyses were performed with the Microbiome Analyst pipeline after total sum scaling (Dhariwal et al., 2017) or Galaxy server^3^.

## Results

### Acetate-Driven Carbon Flow

**FIGURE 1:**
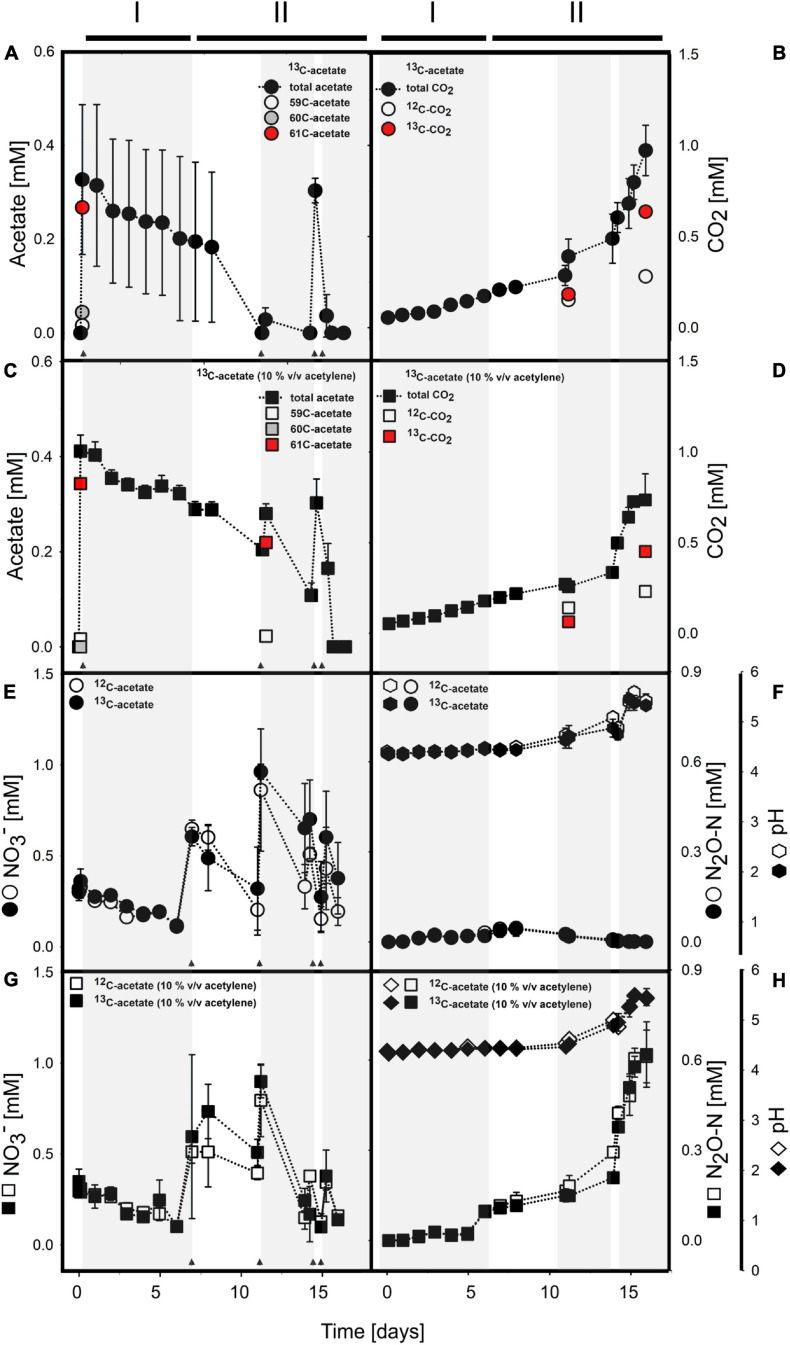
Acetate **(A,C)** and nitrate **(E,G)** consumption concomitant to CO_2_ **(B,D)** and N_2_O **(F,H)** production in anoxic incubations of acetate supplemented cryoturbated peat circle sediments without **(A,B,E,F)** and with **(C,D,G,H)** acetylene. pH is shown in panels **(F)** and **(H)**. Arrows indicate time of acetate or nitrate supplementation. Shaded and non-shaded areas highlight different periods of nitrate consumption, and lines above the graphs indicate phases (see Table 2). **(A–D)** show ^13^C-acetate treatments only. For details on acetate and CO_2_ in ^12^C-acetate treatments, please refer to Supplementary Figure S1. **(E–H)** include data of ^13^C- and ^12^C-acetate treatments as indicated in the insert. 59C-acetate, unlabeled ^12^C_2_-acetate; 60C-acetate, half-labeled ^13^C_1_-acetate; and 61C-acetate, fully labeled ^13^C_2_-acetate. Values are means ± standard deviation of triplicate incubations except for isotope analyses where 1-3 values were obtained (see text for details).

Acetate was below the detection limit (i.e., <20 μM) in anoxic cryoturbated peat circle sediment incubations prior to supplementation. Initially supplemented acetate was consumed without apparent delay and primarily converted to CO_2_ in all treatments (Figures 1A–D, Table 1, and Supplementary Figure S2). Traces of CH_4_ were detected, suggesting a marginal role of methanogenesis for carbon and electron flow (Supplementary Figure S3). Acetate was refed after day 11, when initial acetate was depleted in treatments without acetylene, and on days 14 as well as 15 (Figures 1A,C). Acetate consumption accelerated with incubation time. Acetate and CO_2_ concentrations from the different treatments were similar per timepoint (*p* > 0.05, ANOVA). 0.72 to 1.01 mM acetate were supplemented in total by the end of the incubation. Carbon recoveries based on total C-flow suggest that 56 and 83% of acetate carbon was oxidized to CO_2_ during phase I (6 days) and II (9 days), respectively (Figure 1 and Table 2), the non-recovered acetate carbon of 27–44% being indicative of assimilation.

**TABLE 1:** Cumulated supplementation of acetate and nitrate, and cumulated production of carbon dioxide and nitrous oxide during incubations in treatments with and without acetylene.

	Without acetylene		With acetylene	
Compound [mM]	^12^C-acetate	^13^C-acetate	^12^C-acetate	^13^C-acetate
CO_2_	1.01 ± 0.03	0.92 ± 0.09	0.73 ± 0.02	0.72 ± 0.02
Acetate	0.66 ± 0.06	0.62 ± 0.16	0.46 ± 0.16	0.54 ± 0.03
NO_3_^–^	1.59 ± 0.10	1.62 ± 0.21	1.54 ± 0.04	1.61 ± 0.03
N_2_O	0.00 ± 0.00	0.00 ± 0.00	0.32 ± 0.04	0.31 ± 0.05

*Mean values of technical replicates with standard deviation.*

**TABLE 2:** Mass- and electron balances during anoxic incubations of cryoturbated peat circle sediments in the presence of supplemental acetate and nitrate (see Figure 1).

Phase I (days 0–6)						Phase II (days 6–15)					
				Recovery (%)						Recovery (%)	
Consumption (mean ± SD μM)		Production (mean ± SD μM)		C- or N-mol	e^–^	Consumption (mean ± SD μM)		Production (mean ± SD μM)		C- or N-mol	e^–^
Δ Acetate	11119	Δ CO_2_	12413	56	n.a.	Δ Acetate	405172	Δ CO_2_-C	669186	83	n.a.
		Δ CH_4_	23	0.8	1.5		1,148186	Δ CH_4_-C	02	0	0
Δ NO_3_^–^	21547	Δ N_2_O-N	2510	12	21	Δ NO_3_^–^-N	4015	Δ N_2_-N	49890	42	93
		Δ N_2_-N	7111	33	72	Δ N_2_O-N	77			n.a.	1.5
		ΔNH_4_^+^-N	85	4	13	Δ NH_4_^+^-N				n.a.	n.a.
				C_*tot*_^*a*^ 57	e^–^_*tot*_^*b*^					C_*tot*_ 83	e^–^_*tot*_
				N_*tot*_^*b*^ 50	107					N_*tot*_ 42	95

*Values for acetate, CO_2_, CH_4_, and NH_4_^+^ represent means ± SD of all incubations. Values for N_2_O are based on treatments without acetylene, and N_2_ values were calculated by subtracting N_2_O from treatments without acetylene from those with acetylene. Electron (e^–^) release was calculated based on CO_2_ production (i.e., dissimilation assuming complete acetate oxidation) rather than acetate consumption that represents assimilation and dissimilation.^*a*^C_*tot*_, total recovery of acetate carbon in CO_2_ and CH_4_.^*b*^e^–^_*tot*_, total recovery of electrons released during dissimilation of acetate, i.e., oxidation to CO_2_, in reduced N-species.^*c*^N_*tot*_, total recovery of nitrogen in N_2_O, N_2_, and NH_4_^+^.*

Mean proportions relative to total acetate of ^13^C_2_- (m/z = 61; fully labeled) and ^13^C_1_-acetate (m/z = 60) in ^13^C-acetate treatments at day 1 were 82.6 ± 1.2% and 12.9 ± 0.5%, respectively, suggesting a moderate portion of ^12^C- in treatments with ^13^C_2_-acetate (Figure 1A). Proportions were essentially the same for ^13^C-acetate treatments with acetylene, i.e., 78.3 ± 2.0% and 13.6 ± 7.9.0 for ^13^C_2_- (m/z = 61; fully labeled) and ^13^C_1_-acetate (m/z = 60), respectively (Figure 1C). Such proportions were the same at day 11 when ^13^C_2_- and ^13^C_1_-acetate was spot checked. At the end of incubation, acetate values were near the detection limit. Thus, the majority of data for isotopic composition of acetate in ^13^C-acetate treatments were not obtained. One replicate, however, showed an ^13^C_2_-acetate proportion of 99.6%. Volatile organic acids other than acetate representing typical fermentations products like propionate and butyrate were negligible (<1 μM) during and after incubation, hence ^13^C-labeled carbon in organic acids was not detectable. ^12^C_2_-acetate (m/z = 59; unlabeled) proportions of total acetate in ^12^C-acetate treatments after initial supplementation was 98.7 ± 1.9%, representing the natural proportion of the ^13^C-isotope. Acetate consumption and CO_2_ production in ^12^C-acetate treatments were highly similar to those in ^13^C-acetate treatments (Figures 1A,C, Table 1, and Supplementary Figure S2). Means from treatments irrespective of acetylene supplementation of acetate consumption rates (in μM day^–1^) were 17.92 ± 22.92 and 17.25 ± 20.42 for ^13^C- and ^12^C-acetate treatments, respectively, during phase I. Corresponding CO_2_-production rates (in μM day^–1^) were 20.42 ± 1.92 and 21.00 ± 2.17 for ^13^C- and ^12^C-acetate treatments, respectively. Acetate consumption and CO_2_ production rates increased during phase II and ranged from 15 to 530 μM day^–1^ for all treatments per re-feeding. Mean ^13^C- and ^12^C-acetate consumption rates of all treatments irrespective of acetylene supplementation during phase II were 173 ± 192 and 186 ± 224 μM day^–1^, respectively.

^13^CO_2_ was spot checked at days 11 and 16 (Figures 1B,D). CO_2_ of ^13^C-acetate treatments without and with acetylene had mean ^13^C-proportions of 54.7 ± 1.4%, and 31.1 ± 0.4%, respectively, at day 11. At the end of incubation at day 16, the mean ^13^C-CO_2_ abundance in ^13^C-acetate treatments without acetylene was 69.4 ± 3.4%. For ^13^C-acetate treatments with acetylene, only one replicate was measured with an ^13^C-proportion of 66.2%.

### Acetate-Driven Electron Flow and pH

Acetate consumption was concomitant to consumption of endogenous nitrate without apparent delay (Figure 1). Endogenous nitrate was depleted within 6 days of incubation, which was similar in all treatments (phase I; Figures 1E,G and Table 2). Nitrate was the most abundant endogenous inorganic electron acceptor detected in cryoturbated peat sediments and approximated 300 μM (Figures 1E,G). Sulfate and iron(II) were almost always below the detection limit in all treatments, suggesting that sulfate and iron respiration were negligible for electron flow. Initial nitrate consumption was concomitant to the production of minor amounts of N_2_O in the absence of acetylene in phase I when the pH was stable at *in situ* levels of 4.4 (Figures 1E,F). Larger quantities of N_2_O were produced in the presence of acetylene (Figures 1E–H). Minor amounts of NH_4_^+^ in the μM range were likewise produced during phase I (Supplementary Figure S4). Recovery of nitrate N in N_2_-N was approximately 3- and 8-fold higher than in N_2_O and ammonium, respectively, at the end of phase I (Table 2). When nitrate was refed upon first depletion to concentrations reflecting endogenous levels (phase II), N_2_O was consumed together with nitrate and N_2_ was the primary reduced end product (Figures 1E–H and Table 2). Electron flow to N_2_ accounted for 72 and 93% of electrons released during acetate oxidation to CO_2_ after phase I and II, respectively. Mean N_2_O to (N_2_ + N_2_O) ratios were 92% during the first days of phase I, and dropped to 25 and <1% from days 6–13 and 14–18 during phase II, respectively. The pH remained in the acidic range but increased from 4.4 to 5.5 during phase II from day 11–14 onward, which was concomitant to an increase in N_2_O production and consumption in treatments with and without acetylene, respectively (Figures 1F,H). Indeed, the pH was significantly different from the start of incubation from day 11 and 14 on in treatments without and with acetylene, respectively (*p* < 0.05, ANOVA). Nitrate concentrations in all treatments were similar during the incubation period (*p* > 0.05, ANOVA). A total of about 1.5 mM NO_3_^–^ was consumed during the incubation time (Table 1).

### Stable Isotope Probing of Bacterial and Archaeal 16S rRNA

16S rRNA-SIP was applied to trace and identify Bacteria and Archaea thriving under nitrate reducing conditions, i.e., putative nitrate reducers and denitrifiers, which are capable of ^13^C-acetate assimilation in acidic peat circle sediments. DGGE of heavy and light fractions showed visible differences in banding patterns, suggesting distinct community composition in “heavy” and “light” 16S rRNA fractions, and successful ^13^C-labeling of ^13^C-acetate assimilating bacteria during incubation (Supplementary Figure S5).

**FIGURE 2:**
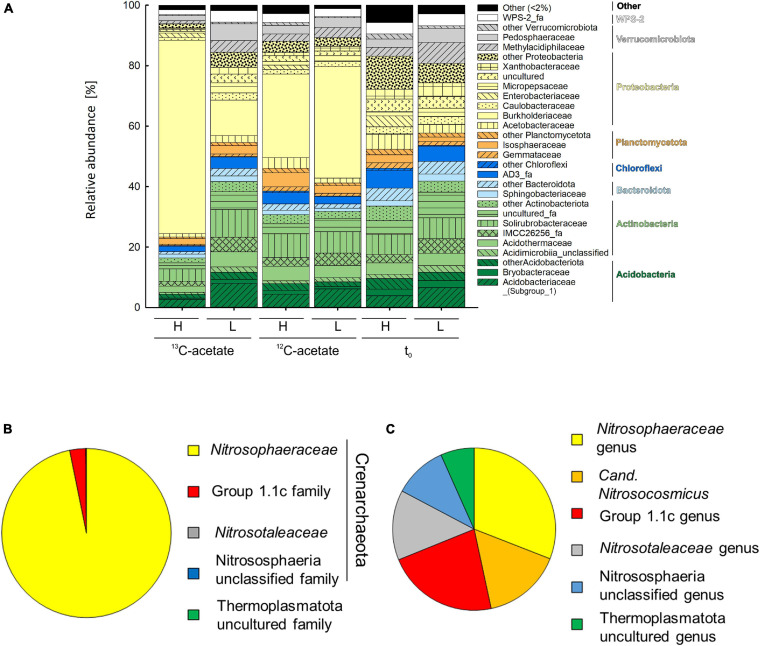
Mean relative abundance of bacterial families (>2% of relative abundance in at least one sample, **A**) delineated from analysis of density resolved 16S rRNA sequences retrieved from cryoturbated peat circle sediments prior to and after 16 days of anoxic incubation (see Figure 1). Values are means of triplicate incubations. Heavy (H) and Light (L) indicate fractions representing relative abundances of ^13^C-labeled and unlabeled 16S rRNA sequences after isopycnic centrifugation (see section “Materials and Methods” for further details) where sequences have been retrieved from. ^13^C- and ^12^C-acetate refer to treatments with ^13^C- and ^12^C-acetate, respectively. *t*_0_ indicates peat circle sediments prior to incubation. Relative abundances in amplicon libraries of archaeal family **(B)** and genus **(C)** level taxa in all samples are displayed. Please note that Log_10_ of sequence counts per genus are given to highlight rare genera **(C)**. For a more detailed overview of archaeal taxa see Supplementary Figure S7.

An average of 26,152 ± 9,481 sequences and a mean Good’s coverage (Good, 1953) of 95.64 ± 0.9 % per sample were obtained for the bacterial communities characterized in triplicates. In total 9,923 genus-level OTUs (97% criterion) were retrieved. Alpha diversity measured by the Inverse Simpson index that covers both richness and evenness were highest for samples before incubation (Table 3). Archaeal communities characterized in triplicates (except for *t*_0_ heavy fraction, from which only two samples were obtained) had an average of 12,590 ± 6,669 sequences among all samples and a mean coverage of 96.4 ± 0.6% per sample. 6,818 archaeal OTUs were retrieved. Inverse Simpson indices of Archaea were lower than those of Bacteria for all samples (Tables 3, 4). The phyla Actinobacteriota (24.0 ± 2.4% in heavy and 30.2 ± 1.3% in light fraction) and Proteobacteria (30.8 ± 2.0% in heavy and 23.0 ± 2.1% in light fraction) with associated families were most prominent prior to incubation (Figure 2A and Supplementary Figure S6). Interestingly, *Rhodanobacteraceae* of the Gammaproteobacteria were detected at an overall relative abundance of 3‰. The classes Acidimicrobiia (8.6 ± 0.4% in heavy, 15.5 ± 0.3% in light fraction), Actinobacteria (7.7 ± 1.6% in heavy, 6.7 ± 0.3% in light fraction), Thermoleophilia (7.6 ± 0.6% in heavy and 8.0 ± 1.2% in light fraction), Alpha- and Gammaproteobacteria (19.1 ± 1.6% and 11.7 ± 0.9% in heavy and 19.9 ± 2.0% and 3.2 ± 0.1% in light fraction, respectively) of the two dominant phyla were prevalent (Supplementary Figure S6). Archaeal communities consisted almost exclusively of sequences affiliating with the class Nitrososphaeria of the phylum Crenarchaeota (former Thaumarchaeota). Nitrososphaeria included unclassified sequences, *Nitrososphaeraceae*, *Nitrosotaleaceae*, and Group 1.1c related sequences (Figures 2B,C and Supplementary Figure S8) and dominated with a relative abundance of 99.99 ± 0.01% across all treatments the overall archaeal community. Only one OTU was classified to the genus level, i.e., Cand. *Nitrosocosmicus* that accounted for <0.5% of all archaeal sequences. Interestingly, typical methanogenic Euryarchaeota were not detected. Thermoplasmatota related sequences (one OTU only) rarely occurred and accounted for 0.01% of sequences from all samples (Figures 2B,C). This OTU was classified as member of the Thermoplasmata and showed 86% 16S rRNA identity to *Methanomassilicoccus luminyensis* B10 (NR_118098.1). Most archaeal sequences were generally only distantly related to described species, highlighting the high degree of phylogenetic novelty of peat circle Archaea. Archaeal communities were essentially stable during the incubation and similar between treatments (Supplementary Figure S8).

**TABLE 3:** Inverse Simpson values for bacterial 16S rRNA amplicon sequences. Mean values of biological replicates with standard deviation.

Treatment	Fraction	Inv Simpson^*a*^	LCI^*b*^	HCI^*b*^
*t*_0_^*c*^	Heavy	134.7 ± 7.6	129.0 ± 7.3	140.9 ± 7.9
*t*_0_	Light	100.2 ± 6.4	96.0 ± 6.4	104.8 ± 6.4
^12^C-acetate	Heavy	12.9 ± 3.4	12.3 ± 3.2	13.6 ± 3.7
^12^C-acetate	Light	7.5 ± 2.1	7.2 ± 2.0	7.8 ± 2.2
^13^C-acetate	Heavy	2.5 ± 0.1	2.4 ± 0.1	2.5 ± 0.1
^13^C-acetate	Light	48.1 ± 3.1	45.6 ± 2.9	50.8 ± 3.3

*^*a*^Larger values indicate higher α-diversity.^*b*^LCI and HCI indicate the 95% low- end and high-end confidence intervals, respectively.^*c*^*t*_0_ – before incubation, all other samples at end of incubation.*

**TABLE 4:** Inverse Simpson values for archaeal 16S rRNA amplicon sequences. Mean values of biological replicates with standard deviation.

Treatment	Fraction	Inv Simpson^*a*^	LCI^*b*^	HCI^*b*^
*t*_0_^*c*^	Heavy	1.1 ± 0.0	1.1 ± 0.0	1.1 ± 0.0
*t*_0_	Light	1.2 ± 0.0	1.2 ± 0.0	1.2 ± 0.0
^12^C-acetate	Heavy	1.1 ± 0.0	1.1 ± 0.0	1.1 ± 0.0
^12^C-acetate	Light	1.2 ± 0.0	1.2 ± 0.0	1.2 ± 0.0
^13^C-acetate	Heavy	1.1 ± 0.0	1.1 ± 0.0	1.2 ± 0.0
^13^C-acetate	Light	1.2 ± 0.0	1.2 ± 0.0	1.2 ± 0.0

*^*a*^Larger values indicate higher α-diversity.^*b*^LCI and HCI indicate the 95% low- end and high-end confidence intervals, respectively.^*c*^*t*_0_ – before incubation, all other samples at end of incubation.*

In contrast, bacterial community structure changed during incubation, which was reflected in the dominant phyla retrieved after incubation. Bacterial communities were dominated by Proteobacteria primarily consisting of Gammaproteobacteria, with relative abundances of 31.5 ± 3.9 and 38.4 ± 4.7 in heavy and light fractions of ^12^C-acetate treatments, respectively, and relative abundances of 66.4 ± 2.7 and 14.7 ± 0.4 in heavy and light fractions of ^13^C-acetate treatments, respectively (Figure 2A and Supplementary Figure S7). OTU 1, associated with the *Burkholderia-Caballeronia-Paraburkholderia* (*Burkholderiaceae*; Burkholderiales; former Betaproteobacteriales) dominated in both ^13^C- and ^12^C-acetate treatments with relative abundances of up to 91% of all Gammaproteobacteria after 16 days of incubation, compared to a relative abundance of only about 1% of all Gammaproteobacteria before incubation (Figure 2A and Supplementary Figure S7). Such data suggest a strong enrichment of Gammaproteobacteria during incubation.

**FIGURE 3:**
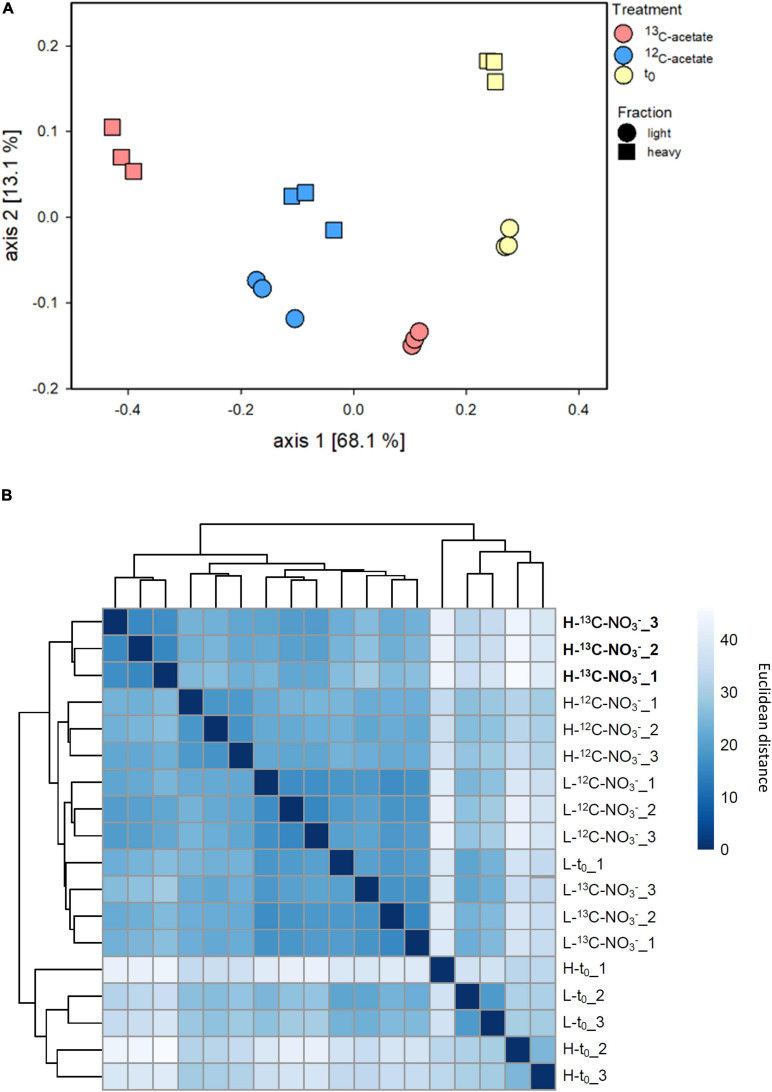
Principal coordinates analysis (PCoA; **A**) based onBray–Curtis dissimilarity calculated from relative abundance data of bacterial species-level OTUs from density resolved 16S rRNA sequences retrieved from cryoturbated peat circle sediments prior to (*t*_0_) and after 16 days of anoxic incubation (see Figure 1). Hierarchical heat map clustering is presented in panel **(B)**. Color code indicates levels of dissimilarity. Sample code: H and L indicate heavy and light fractions, respectively; *t*_0_, ^13^C- and ^12^C- represent peat circle sediments prior to incubation, ^13^C- and ^12^C-acetate treatments, respectively. Numbers are indicative of replicate. Heavy fractions of treatemnts with labelled acetate are printed in bold.

Density resolved bacterial communities after and prior to the incubation differed, while the replicates showed high similarities (Figure 3 and Supplementary Figure S7A). Differences in bacterial β-diversity evaluated by ANOVA did not differ significantly by replicates (*p* = 0.4871) but were different by treatment and fraction (*p* < 0.001). This was supported by principal coordinate analysis (PCoA), which revealed clustering by replicate and fraction of treatments (Figure 3A). The PCoA plot shows a clear separation on axis 1 explaining 68.1% of variability, and separates ^13^C-acetate heavy fractions from the associated light fractions, as well as from ^12^C-acetate treatments and *t*_0_ samples obtained prior to incubation. Heavy and light fractions of ^12^C-acetate control treatments clustered together with light fractions of ^13^C-acetate treatments (Figure 3B). Most communities retrieved prior to incubation (*t*_0_) formed a distinct cluster (Figure 3B). Heavy fractions from ^13^C-acetate treatments likewise showed a distinct clustering pattern, supporting successful ^13^C-labeling of 16S rRNA. PCoA did not show clear trends for Archaea (Supplementary Figure S9) with neither treatments, nor fractions clustering together. ANOVA revealed no significant difference between replicates (*p* = 1) or treatments and fractions (*p* > 0.091–0.714).

**FIGURE 4:**
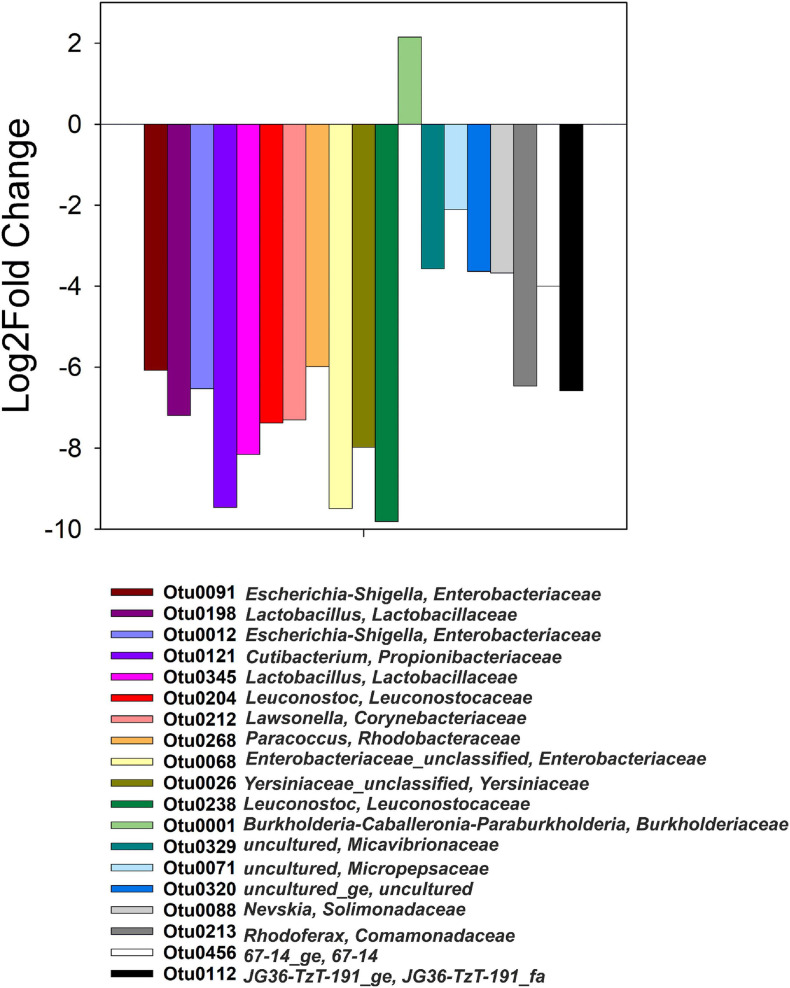
Genus-level taxa with significantly different relative abundances (*p* < 0.05) in heavy and light fractions of ^13^C-acetate treatments only, i.e., such taxa were not differentially abundant in heavy than light fractions at *t*_0_ or ^12^C-acetate treatments.

**FIGURE 5:**
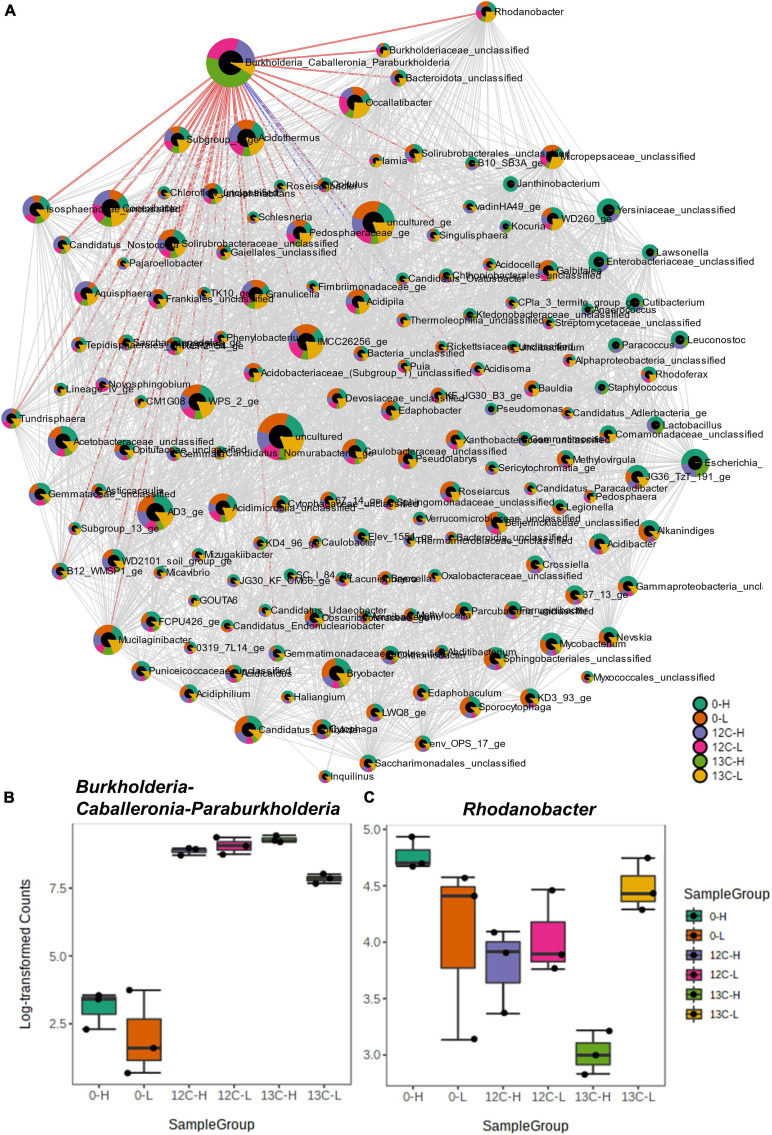
Correlation network analysis of density resolved 16S rRNA sequences retrieved from cryoturbated peat circle sediment incubations (see Figure 1) using the SparCC algorithm **(A)**. Nodes represent genus-level taxa, and red and blue edges represent positive and negative correlations of labeled OTU 1 (*Burkholderia-Caballeronia-Paraburkholderia*). For more details on correlations please refer to Supplementary Table S1. Node size reflects taxon abundance and colors indicate the relative proportion per sample. The box plots **(B,C)** show the abundances of *Burkholderia-Caballeronia-Paraburkholderia* (B) and of one positively correlated taxon (*Rhodanobacter* spp.; **C**). Sample code: H and L indicate heavy and light fractions, respectively; 0, ^13^ C-, and ^12^C- represent peat circle sediments prior to incubation, ^13^C- and ^12^C-acetate treatments, respectively.

Differential abundance values (Log2Fold change; *p* < 0.05) were computed between heavy and light fractions of each treatment and compared across all treatments (Figure 4). The only OTU that was significantly more abundant in the heavy fractions of ^13^C-acetate treatments compared to those of *t*_0_ samples and those of ^12^C-acetate treatments was OTU 1 affiliating with Burkholderiales (*Burkholderiaceae, Burkholderia-Caballeronia-Paraburkholderia* group). OTU 1 representatives showed 99% identity to 16S RNA genes of *Paraburkholderia ginsengisoli* NBRC 100965. Correlation network analysis indicated a positive interaction of *Burkholderiaceae* OTU 1 with many genus level taxa including *Rhodanobacter* spp. (98 and 96% identity of OTU 70 to *Rhodanobacter ginsengisoli* GR17-7, NR_044127, and *Rhodanobacter denitrificans* 2APBS1, NR_102497.1, respectively), and OTUs affiliating with *Isosphaeraceae* (95% identity of OTU 44 to *Singulisphaera rosea*, NR_116969.1), *Solirubrobacteraceae* (95% identity of OTU 3 to *Conexibacter arvalis* KV-962 NR_113264.1), as well as *Gemmatimonadaceae* (91% identity of OTU 447 to *Gemmatimonas aurantiaca* T-27, NR_074708; Figures 2A, 5A and Supplementary Table S1). The relative abundance of OTU 1 increased by several orders of magnitude in amplicon libraries during incubation and represented a dominant genus at the end of incubations (Figure 5B), and *Isosphaeraceae* (Planctomycetes) related OTUs were the second most important taxa in terms of increase in relative abundance during incubation (Figure 2A). In total, 35 positive and 5 negative correlations were observed for OTU 1 (Figure 5A and Supplementary Table S1). *Rhodanobacter* spp. were present in cryoturbated peat sediments prior to incubation, less affected by incubation than OTU 1, and showed 56 positive and 12 negative correlations with e.g., *Burkholderiaceaea*, *Occallatibacter*, *Iamia*, and *Tundrisphaera* (Figures 5A,C and Supplementary Table S1). Abundances of some archaeal OTUs were significantly different between heavy and light fractions within treatments as judged by Log2FoldChange, but did not show differences between ^13^C- and ^12^C-acetate treatments, demonstrating that Archaea did not assimilate significant amounts of acetate carbon.

## Discussion

### Complete Denitrification in Acidic Peat Circle Sediment Microcosms

Cryoturbated peat circles of the subarctic tundra with an *in situ* pH 4 emit high amounts of N_2_O, which is in the range of heavy fertilized agricultural and tropical rainforest soils (Potter et al., 1996; Werner et al., 2007; Repo et al., 2009). Despite this high propensity to emit N_2_O *in situ*, and an abundant as well as an unusual, hitherto poorly described denitrifier community associated with anaerobic N_2_O production (Palmer et al., 2012), we demonstrated the capacity of the peat circle community to perform complete denitrification rather than nitrate ammonification at pH 4.4 with only minor intermediary accumulation of N_2_O (Figure 1, Table 2, and Supplementary Figure S4). Such an N_2_O consumption at low pH is in line with the previous detection of *nosZ* in peat circle sediments, evidence from peatlands suggesting ongoing N_2_O consumption at low pH, and thus the emerging view that acidic peatlands might temporarily act as a sink for N_2_O (Chapuis-Lardy et al., 2007; Kolb and Horn, 2012; Palmer et al., 2010, 2012). Thus, N_2_O reduction in acidic peat lands including cryoturbated peat circles is a possible mitigator of greenhouse gas emissions at low pH.

### Regulation of Denitrification Associated Net N_2_O Production

Peat circles show a remarkably high total denitrification capacity at *in situ* pH and *in situ* near nitrate concentrations, which is much higher than that of many other peatlands, including pH neutral ones, suggesting an acid-tolerant denitrifier community (Figure 1; Palmer et al., 2010, 2012; Palmer and Horn, 2012, 2015). Acid-tolerant denitrifier communities and N_2_O consumption in situ at pH <6 were previously shown for other peat lands, demonstrating that our findings are in line with previous studies (e.g., Chapuis-Lardy et al., 2007; Palmer et al., 2010; Kolb and Horn, 2012). The increase in N_2_O consumption and denitrification rate in phase II of peat circle microcosms was associated with an increase in pH to 5.5, suggesting that the optimal pH for complete denitrification is higher than the *in situ* pH (Figure 1 and Table 2). Nevertheless, this is a remarkable finding that is in stark contrast to the well-studied neutrophilic model denitrifiers like *Paracoccus denitrificans* that accumulate N_2_O when pH falls below 7, and suggests that strains capable of N_2_O-reduction at pH <6 are more important than previously thought (Bergaust et al., 2010; Liu et al., 2014; Lycus et al., 2017). Evidence for microbial N_2_O consumption at low pH comes from few isolates of the genus *Rhodanobacter* capable of N_2_O reduction at pH 4 to 5.7, and the detection of *Rhodanobacter* sp. in this study (Figure 5C; Van Den Heuvel et al., 2010; Prakash et al., 2012; Lycus et al., 2017). Notably, one of such strains reduced N_2_O to N_2_ at pH 5.7, but not at more neutral or acidic pHs tested, suggesting the existence of a small ecological niche in terms of pH optimum for N_2_O reduction for certain acid-tolerant N_2_O reducers The formation of more pH neutral microsites in local denitrification “hot spots” during peat storage or during microcosm incubations, where functional N_2_O reductases of “classical” denitrifiers might have developed represent alternative explanations for the observed N_2_O reduction capacities at low overall pH. However, although the existence of such pH-neutral “hot spot” microsites cannot be fully excluded, electron flow to N_2_O was of minor importance during the first day of incubation, and substantially increased during the course of the 16 day incubation (Figure 1 and Table 2). Such findings rule out the existence of large amounts of functional N_2_O reductases at the beginning of the incubation, i.e., at the end of the storage period. Regular shaking of microcosms and a high buffering capacity of peat material as indicated by the stable pH during phase I (Figures 1F,H) minimize the likelihood for the occurrence of pH neutral “hot spot” microsites during our incubations. Notably, the peat material is static *in situ* during the growing season, and has thus a higher probability than during our incubations to form pH neutral microsites, where classical functional N_2_O reductases might develop. However, large quantities of functional N_2_O reductases were absent as evidenced by the little initial electron flow to N_2_O in the peat material and high *in situ* N_2_O emission rates (Table 2 and Figure 1; Marushchak et al., 2011; Palmer et al., 2012). Thus, it is more likely that the observed N_2_O reduction at pH <6 can be explained by the existence of a truly acid-tolerant or acidophilic N_2_O-reducer community, rather than by the formation of more pH neutral microsites by clustering of active denitrifiers.

Consequently, low pH as a reason for the impairment ofN_2_O reduction *in situ* and a regulation of N_2_Ofluxes appears to be of minor importance for certain low pHenvironments. Interestingly, Palmer et al. (2012) investigated apparentMichaelis-Menten kinetics of nitrate-dependent denitrification inanoxic microcosms and found that peat circle denitrifiers weresaturated with less than half of the NO_3_^–^ concentrations occurring *in situ*, and suggested a limitationof electron donor availability that restricts denitrification incryoturbated peat circles (Palmer et al., 2012). Expression of notableN_2_O reductase activity occurred at the end of phase I in ourexperiments (Figure 1), suggesting thatN_2_O consumers were stimulated by the incubation in thepresence of supplemental acetate. Regulation of N_2_O reduction by the availability of electron donors is well known for denitrifiers like the bacterial denitrifier *Alcaligenes faecalis*. *A. faecalis* immediately reduces accumulated NO_2_^–^ and increases N_2_ production while not changing N_2_O production upon pulses of the electron donor acetate in steady state culture (Schalk-Otte et al., 2000). The increase of ^13^C_2_-acetate derived ^13^CO_2_ proportion from 50 to about 70% over time suggests that substantial amounts of endogenous carbon were utilized along with the supplemental acetate during phase I, and that microbially available endogenous carbon pools became more and more depleted during incubation. ^13^C_2_-Acetate derived ^13^CO_2_ represented most of the total CO_2_ at the end of phase II, suggesting a preferential mineralization (i.e., complete oxidation) of supplemental acetate during denitrification in peat circle sediments toward the end of the incubation. Such an acetate utilization was concomitant to increased N_2_ production, providing a link between acetate consumption and complete denitrification (Figure 1). In contrast, the endogenous carbon appears to support N_2_O production by denitrification *in situ* and is provided by slightly to moderately decomposed peat (H4-5 on the Von Post Scale) at a rather low C/N ratio of 24 (Marushchak et al., 2011). Humified organic matter and plant remnants containing hard to degrade lignocellulose, hemicellulose, polysaccharides, covalently bound amino acids, and aromatic moieties occur in peat and might fuel denitrification after hydrolysis or pre-oxidation (Black et al., 1955; Coulson et al., 1959; Martin and Manu-Tawiah, 1989; Kuder and Kruge, 2001). Due to such hard-to-degrade organic carbon (“slow release electron donors”), it is not surprising that microbial biomass N is substantially lower in the upper peat circles than in adjacent vegetated tundra peat (Voigt et al., 2017a, b). Low availability of electron donors, eventually reflected in a rather narrow C/N ratio, is well known to limit denitrification and favor N_2_O relative to N_2_ production (van Cleemput, 1998; Marushchak et al., 2011). Thus, combined results support the view that a limitation of microbe-available electron donors in cryoturbated peat circle sediments favors the release of N_2_O, despite the demonstrated potential of the microbial community for complete denitrification that might be unleashed by easily available electron donors (Figure 1).

### Electron Flow and Active Archaeal Community Capable of Anaerobiosis

Complete denitrification rather than nitrate ammonification was the primary nitrate respiration pathway as indicated by the N-mass balance (Table 2). Alternative anaerobic respirations other than nitrate respiration were negligible, demonstrating the divergence of electron flow from the classical anaerobic food chain toward methane to denitrification (Table 2). This was in agreement with the absence of typical endogenous fermentation products, classical euryarchaeal methanogens, and a negligible number of Thermoplasmatota affiliated sequences (0.007%) that were distantly related to *Methanomassilicoccus* spp. (Figure 2). *Methanomassilicoccus* spp. represent a new group of recently discovered methylotrophic methanogens that occur in peatlands, suggesting the possibility that related Archaea of the same order were associated with the production of methane traces in peat circles (Borrel et al., 2013; Söllinger et al., 2016). Detection of the second most important group of Archaea represented by group 1.1c Crenarchaeota after 16 days of anoxic incubation suggest anaerobic capabilities of this group. Indeed, group 1.1c prefers low pH environments, was enriched with methane under oxic and anoxic conditions, frequently detected in peatlands, showed glutamate dependent growth, and was not capable of ammonia oxidation, suggesting a heterotrophic, facultative life style (Lehtovirta et al., 2009; Wüst et al., 2009; Bomberg et al., 2010; Schmidt et al., 2015; Weber et al., 2015). Thus, group 1.1c might play a role in cycling of organic nitrogen in peat circles.

### Ammonia-Oxidizing Archaea Rather Than Bacteria as Major Sources of Nitrate

The by far greatest share of archaeal 16S rRNA sequences was represented by Crenarchaeota (former Thaumarchaeota) of the class Nitrososphaeria/family *Nitrososphaeraceae*. Such “Thaumarchaeota” were recently re-integrated into the phylum Crenarchaeota (Parks et al., 2018), and are well-known ammonia-oxidizing Achaea (AOA) that prefer ammonia at low concentration and environments with a low pH of less than 5.5 (De La Torre et al., 2008; Prosser and Nicol, 2008; Gubry-Rangin et al., 2010; Lehtovirta-Morley et al., 2011). AOA in permafrost-affected soils showed a high β-diversity of “Thaumarchaeota,” with niche differentiation of AOA clades following soil moisture and nitrogen content (Alves et al., 2013). N_2_O fluxes from unvegetated (sub)arctic peat soil surfaces in Finland and Siberia were in the range or even higher (76.8 μg N_2_O-N m^–2^ h^–1^) than from managed peatland soils from northern countries and showed a positive correlation with nitrate concentration of soils and *amoA* gene abundance (Siljanen et al., 2019; Hetz and Horn, 2021). Such *amoA* genes mainly affiliated with the Nitrosophaerales clades NS-gamma (NS-γ; distantly related to Nitrososphaera NS-α) and NS-zeta (NS-ζ; *Cand*. Nitrosocosmicus related), which is in agreement with our 16S rRNA data (Alves et al., 2018; Siljanen et al., 2019). Notably, gross nitrification rates were unaffected by inhibition of bacterial nitrification, which is in line with less than 0.01% of 16S rRNA sequences attributed to bacterial nitrifiers (i.e., *Nitrosomonadaceae*) in amplicon libraries in this study. This is well in agreement with the current view that ammonia-oxidizing Archaea are generally wide spread in permafrost affected acidic environments (reviewed in Hetz and Horn, 2021). Potential nitrite oxidizing bacteria (NOB) of the genera *Nitrospira* and *Nitrobacter* (0.008 and 0.001% relative abundance in 16S rRNA derived aplicon libraries, respectively) were detected. The genus *Nitrospira* has been found in low pH as well as cold environments, is widely distributed in (permafrost-)soils (reviewed in Hetz and Horn, 2021 and references therein), and might therefore be associated with nitrite oxidation to nitrate. Vertical fluctuating oxygen gradients in peat circles due to rainfall events, micro-gradients of oxygen across peat aggregates, and cryoturbation fueled mixing of peat might contribute to high *in situ* nitrification activities and transfer of nitrate and/or nitrite to denitrifiers. Indeed, along with the previously discovered generally high nitrification rates at the low *in situ* pHs, these findings highlight the importance of AOA for the provision of nitrite to nitrite oxidizers and/or denitrifiers, and thus as essential contributors to N_2_O fluxes from permafrost affected northern peatlands including cryoturbated peat circles (Siljanen et al., 2019).

### Active Bacterial Community Capable of Anaerobiosis

Investigated bacterial communities from the microcosm experiment revealed high relative abundances of Actinobacteria and Alphaproteobacteria before supplementation and incubation of peat circle sediment. This is congruent with a high relative abundance of *narG* sequences affiliating with Actinobacteria, which clearly dominated the investigated community of nitrate reducers in this environment and accounted for up to 95% of the overall relative sequence abundance (Palmer et al., 2012). Interestingly, sequences affiliating with *Conexibater* sp. (Solirubrobacteraceae) that are known to grow via nitrate respiration, were third most abundant in amplicon libraries (Figure 2A; Seki et al., 2012). Nitrite reductase genes were indicative of Alpha- and Gammaproteobacteria including Rhizobia and *Rhodanobacter* sp. related sequences. Many nitrite reductase genes affiliated with Betaproteobacterales (Seki et al., 2012). Indeed, *Xanthobacteraceae* (Alphaproteobacteria/Rhizobiales/*Rhodoplanes* spp.) and *Burkholderiaceae* (Gammaproteobacteria/Betaproteobacteriales) were frequently detected on 16S rRNA level in cryoturbated peat circle (Figure 2A). Capabilities for dissimilatory nitrate reduction and denitrification are widespread among *Burkholderiaceae* and *Rhodoplanes* spp., suggesting a contribution to nitrate dissimilation and denitrification of such taxa (Oren, 2014; Mariñán et al., 2019). Alphaproteobacteria (mostly uncultured) and Betaproteobacteriales affiliated *nosZ* were retrieved from peat circle sediments, demonstrating the genetic potential of such organisms to consume N_2_O (Palmer et al., 2012). This is in line with the widespread occurrence of such *nosZ I* genes in arctic environments (reviewed in Hetz and Horn, 2021). However, the abundance of typical *nosZ I* genes was low in peat circle sediments, accounting for only 0.002% of 16S rRNA genes, thus suggesting a minor fraction of the bacterial community with the genetic potential for N_2_O reduction (Palmer et al., 2012). During incubation, the bacterial community structure shifted toward the Gammaproteobacteria that became the most abundant class, with sequences affiliating with *Burkholderia-Caballeronia-Paraburkholderia*. Closely related *nosZ* sequences retrieved from a comparable acidic palsa peat site in Finish Lapland were affiliated with *Burkholderia pseudomallei* (Palmer and Horn, 2012). *Burkholderia-Caballeronia-Paraburkholderia* correlated with *Rhodanobacter* sp. that include acid-tolerant nitrite- and N_2_O reducers (Lycus et al., 2017; Figure 5 and Supplementary Table S1), suggesting phylogenetically associated organisms as possible candidates for the reduction of N_2_O to N_2_ in peat circle sediments.

### Key ^13^C-Acetate Assimilators During Complete Denitrification

OTU1 affiliating with *Burkholderia-Caballeronia-Paraburkholderia* represented the only, highly competitive acetate assimilator during denitrifying conditions as indicated by ^13^C-acetate SIP (Figure 4) that was likewise enriched on total RNA level (Figure 2A). Cross-feeding by ^13^C-labeled substrate derived intermediates of substrate degradation, heterotrophic and autotrophic ^13^CO_2_ fixation as well as mineralization of ^13^C-labeled decaying microorganisms that primarily consumed the ^13^C-labeled substrate are well known issues when applying stable isotope probing (Neufeld et al., 2007). However, the use of low concentrations of essentially non-fermentable ^13^C_2_-acetate, the absence of organic acids like propionate and butyrate that might eventually be produced via carboxylation or condensation of acetate, respectively, intracellular metabolism of acetate via the tricarboxylic acid cycle, recoveries of app. 70% of the ^13^C_2_-acetate carbon in ^13^CO_2_, and the clear ^13^C-labeling of only one taxon (rather than a dilution of the label among many taxa) argue in favor of neglectable cross-feeding. Thus, *Burkholderia-Caballeronia-Paraburkholderia* affiliated taxa were important organisms for acetate-dependent carbon flow, primary acetate consumers, and associated with electron flow to nitrate under anoxic conditions in acidic peat circle sediments. Genomes of two new, recently isolated *Caballeronia* strains (*Burkholderiaceae*) originating from peat circles revealed the presence of multiple nitrate reductases, a *nirBD* encoded nitrite reductase, as well as the NO reductase NorV, suggesting that these isolates are nitrate ammonifiers with the capability to detoxify NO (Hetz et al., 2020). Genome sequences of other *Burkholderia* representatives showed the presence of *nosZ* as well as *nirK* genes (Sanford et al., 2012). Isolates from *Sphagnum* mosses that cover a Finnish acidic mire included isolates from *Burkholderia* sp. that have their optimum pH at around 5 and emit N_2_O, when NO_3_^–^ was supplemented during incubation (Nie et al., 2015). All isolates possessed the *narG* gene for nitrate reduction, but amplification of either typical or atypical *nosZ* gene was not successful, suggesting these *Burkholderia* sp. as incomplete denitrifiers. Sequencing of 16S rRNA targeted DGGE bands from overall microbial community incubations of the same *Sphagnum* tissue retrieved from incubations at 15°C that showed N_2_O production, revealed *Burkholderia* sp. as major representatives of the bacterial community (Nie et al., 2015). Although associated with *Burkholderiaceae* via co-occurrence network analysis (Figure 5A), *Rhodanobacter* spp. affiliating sequences had low relative abundances in amplicon libraries from heavy fractions of ^13^C_2_-acetate treatments (Figure 5C), which is in agreement with the high relative abundance of *Burkholderiaceae* related sequences (Figure 5B), suggesting that peat circle *Rhodanobacter* spp. were unable to assimilate acetate. Indeed, acetate assimilation is a rare feature among members of this genus (Prakash et al., 2012). Such collective findings demonstrate that organisms of the *Burkholderia-Caballeronia-Paraburkholderia* group are widespread in peatlands, and include competitive, acid-tolerant acetate-consumers coupling acetate consumption to dissimilatory nitrate reduction and/or denitrification in peat circle sediments.

## Conclusion

Acid-tolerant microbes from permafrost-affected cryoturbated peat circle sediment of the Arctic tundra were capable of complete denitrification at pH 4 in the presence of acetate as electron donor, demonstrating that N_2_O reduction was not drastically impaired by the low pH (Figure 1 and Table 2). *Burkholderiaceae* were most competitive acetate assimilators during denitrification, suggesting a prominent role of this taxon in dissimilatory nitrate reduction including denitrification, and demonstrating that very few key taxa can be responsible for most of the activities under certain conditions (Figures 1, 4). *Rhodanobacter* related taxa known for N_2_O-reduction at acidic pH and for the rare ability of members of the genus to assimilate acetate (Prakash et al., 2012; Lycus et al., 2017) co-occurred with Burkholderiaceae, suggesting a trophic inter-species interaction responsible for complete denitrification at acidic pH, e.g., via transfer of nitrite and/or N_2_O (Figure 5). However, peat circles emit large amounts of N_2_O, and increasing temperatures increase permafrost thaw, N-availability and emission of multiple greenhouse gases including N_2_O (Salmon et al., 2016; Voigt et al., 2017a, b). Peat circles are thus prone to respond to global change by increased N_2_O emissions. High denitrification associated N_2_O production potentials of peat circle sediments and other permafrost affected peatlands were previously shown and confirmed in this study, despite the high potential for N_2_O consumption demonstrated in this study (Figure 1; Palmer et al., 2012; Palmer and Horn, 2012). Such a paradoxon might be resolved when the rather low fraction of mineralized endogenous organic carbon is taken into account (Figures 1B,D), suggesting a possible limitation of denitrifiers in accessible electron donors yielding high N_2_O to (N_2_ + N_2_O) ratios due to an impairment of electron flow to N_2_O. More research is certainly needed to address this thesis in future studies.

## Data Availability Statement

The datasets presented in this study can be found in online repositories. Datasets of bacterial and archaeal 16S rRNA gene sequences derived from amplicon sequencing were deposited at the NCBI sequence read archive under BioSample accession numbers SAMN14211851 to SAMN14211856 and SAMN14210576 to SAMN14210581 respectively, in BioProject PRJNA608855.

## Author Contributions

SH and MH designed the SIP experiments, wrote the original manuscript, and interpreted data. SH set up microcosms and performed all laboratory work, if not stated otherwise. MH conceived the original idea and supervised all laboratory work. Both authors contributed to the article and approved the submitted version.

## Conflict of Interest

The authors declare that the research was conducted in the absence of any commercial or financial relationships that could be construed as a potential conflict of interest.

## Footnotes

1 https://www.arb-silva.de/download/arb-files/

2 https://mothur.org/blog/2020/SILVA-v138-reference-files/#legalese

3 https://usegalaxy.eu/
