# Supplementary material for: Burkholderiaceae Are Key Acetate Assimilators During Complete Denitrification in Acidic Cryoturbated Peat Circles of the Arctic Tundra
Source: Front Microbiol. 2021 Feb 5;12:628269. doi: 10.3389/fmicb.2021.628269 (PMC7892595; doi:10.3389/fmicb.2021.628269)
Supplement: Supplementary file 1 [file Data_Sheet_1.pdf]

Figure S1 (appendix)

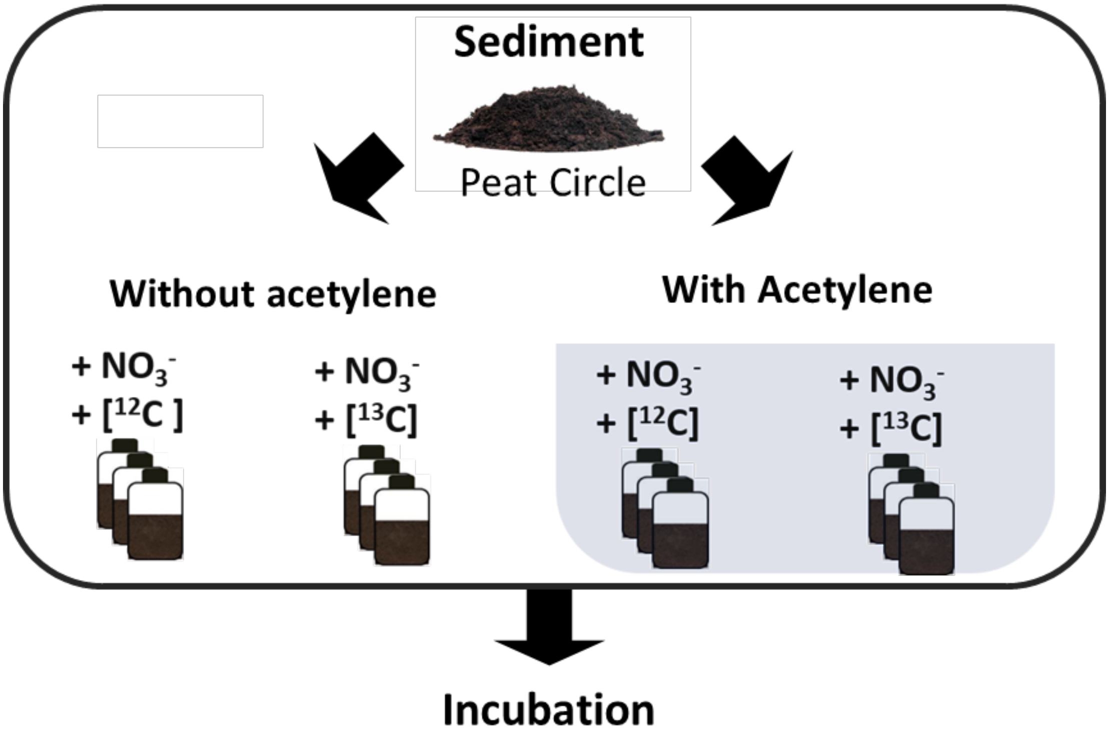

Figure S1. Experimental setup for triplicate anoxic peat circle sediment (pH 4.4) slurries utilized for acetate 16S rRNA stable isotope probing and concomitant analysis of denitrification products and intermediates. Incubations with peat circle sediments containing endogenous nitrate were supplemented with 400 µM of unlabeled [^{12}C]- or double-labelled [^{13}C]-acetate. Two sets of incubation were set up, one with and one without acetylene (10%-v/v) in the headspace. Only incubations without acetylene were used for total RNA extraction and stable isotope probing. Unlabeled [^{12}C]-acetate treatments served as controls for the stable isotope probing approach to identify labeled taxa, i.e. to account for drift of unlabeled RNA into 'heavy' fractions with a characteristic buoyant density of [^{13}C-]-labeled RNA. Acetylene treatments served as process controls to determine denitrification derived N_2 production via the acetylene inhibition method. Nitrate and acetate were re-fed during the course of the incubation upon consumption (see Figure 1). Incubation was at an *in situ* relevant temperature of 15 °C in the dark. Microcosms were rigorously shaken manually twice a day.

Figure S2 (appendix)

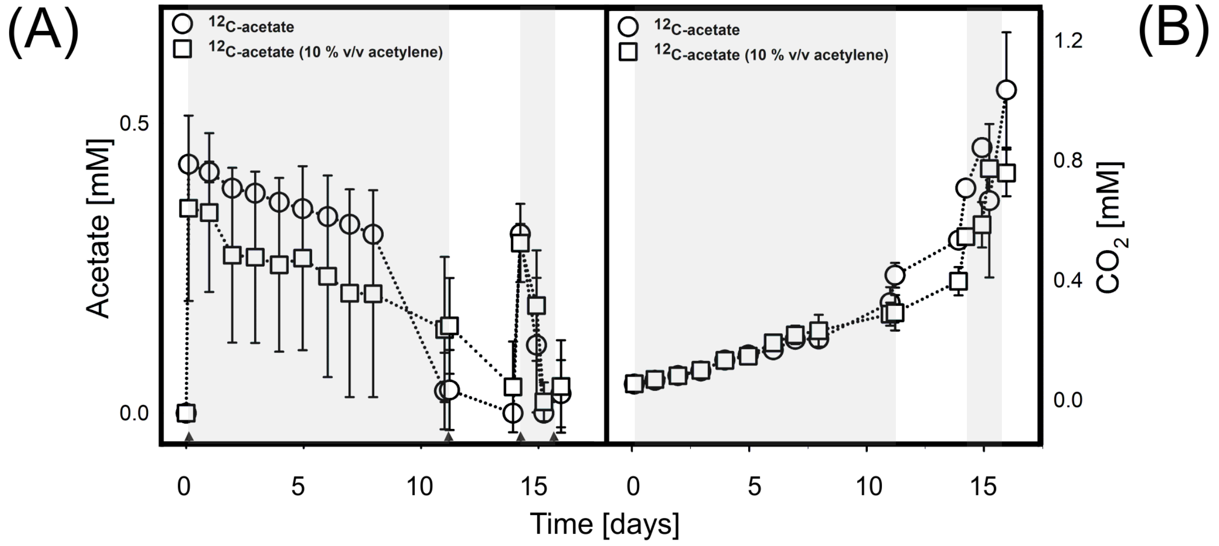

Figure S2. Acetate consumption (A) and CO_2 production (B) in anoxic incubations of ^{12}C-acetate supplemented cryoturbated peat circle sediments without and with acetylene. Arrows indicate time of acetate supplementation. Shaded and non-shadeded areas highlight different periods of nitrate consumption (Figure 1). Values are means ± standard deviation of triplicate incubations.

Figure S3 (appendix)

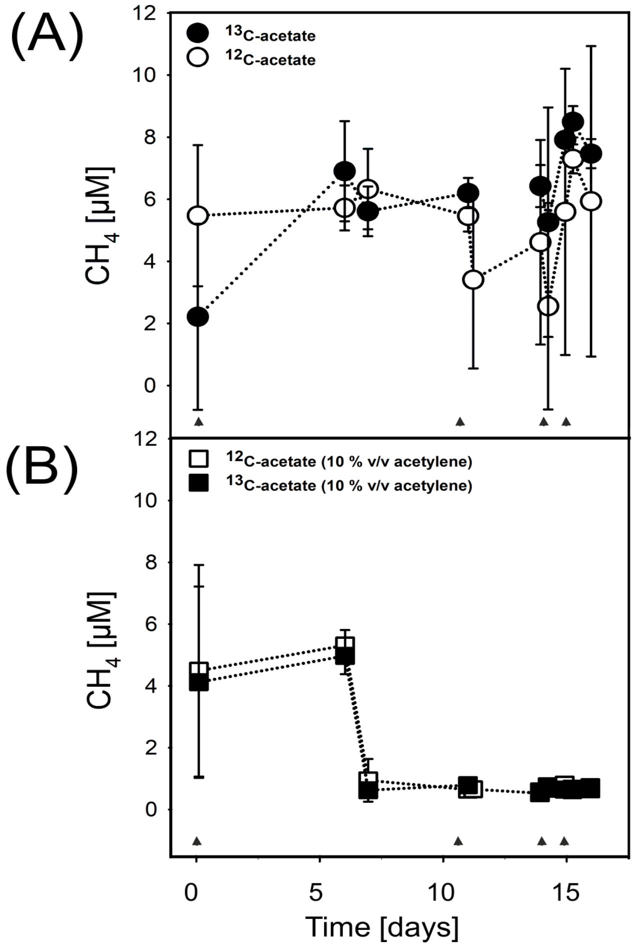

Figure S3. Methane concentrations in anoxic incubations of acetate supplemented cryoturbated peat circle sediments without (A) and with (B) acetylene (Figure 1). Arrows indicate time of acetate supplementation. Values are means ± standard deviation of triplicate incubations.

Figure S4 (appendix)

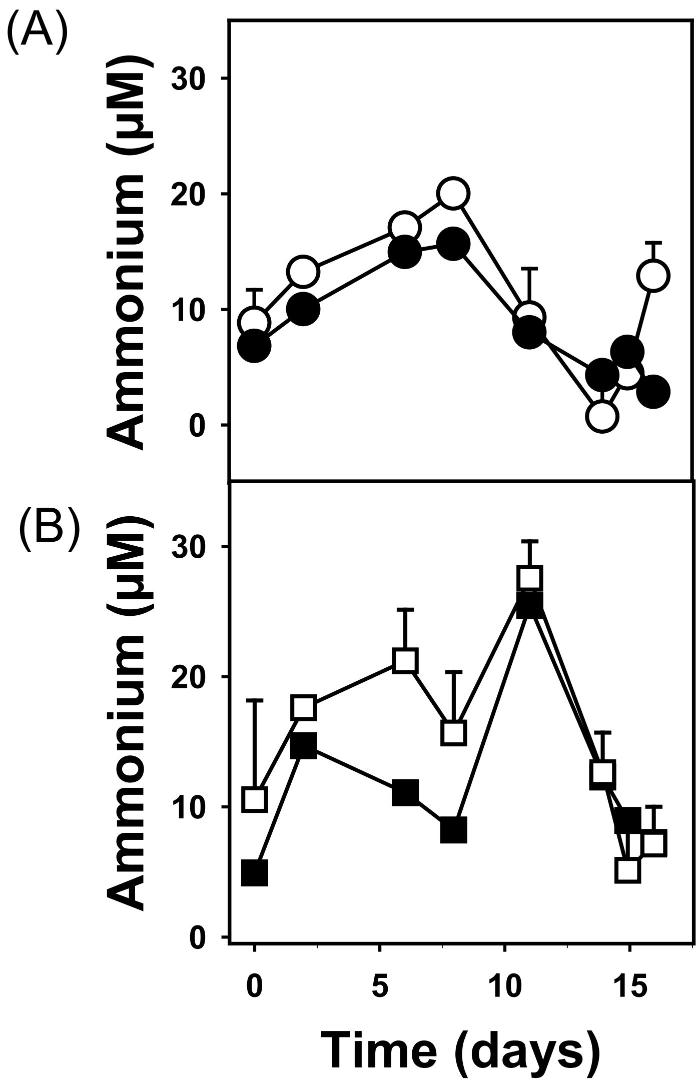

Figure S4. Ammonium concentrations in anoxic incubations of acetate and supplemented cryoturbated peat circle sediments without (A) and with (B) acetylene (Figure 1). Values are means ± standard deviation of triplicate incubations.

Figure S5

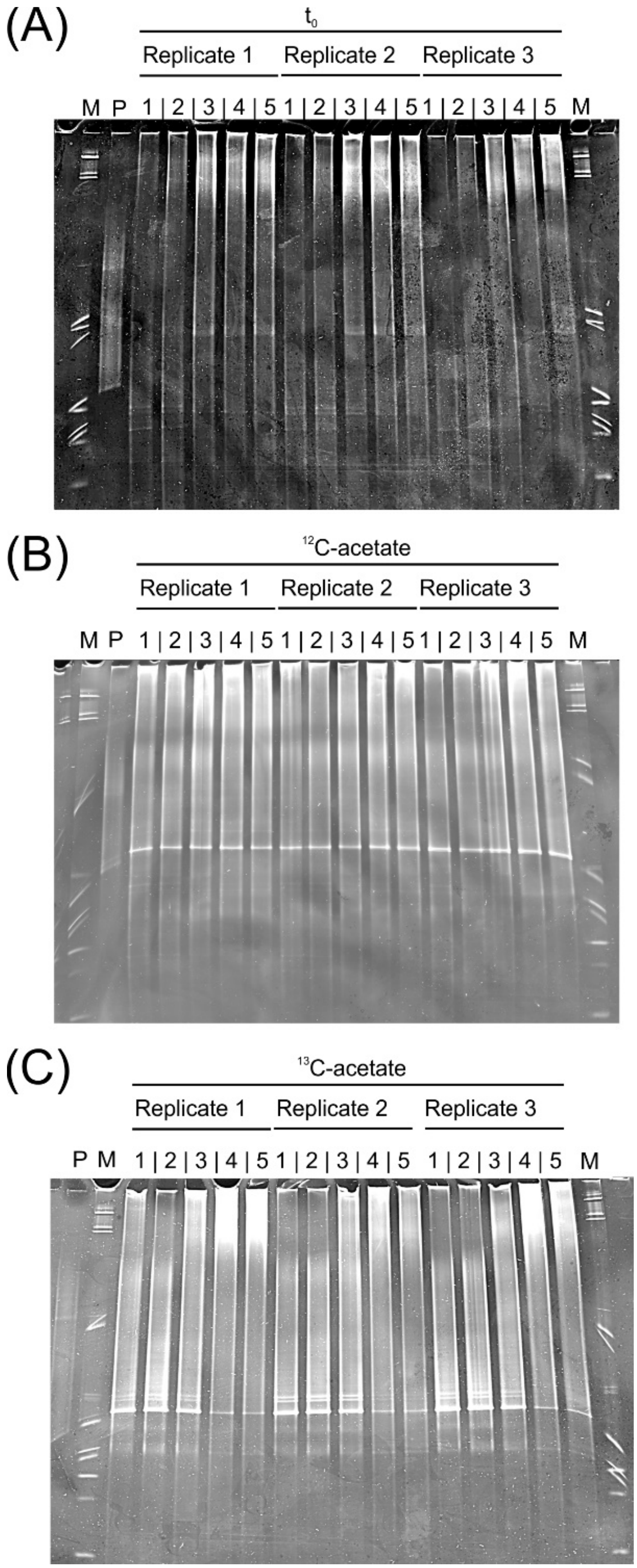

Figure S5. DGGE gels of heavy and light fractions of all replicates (1-3) and treatments. 1 – heaviest fraction; 5 – lightest fraction; M – marker; P – positive control.
A: Heavy and light fractions from all three replicates of t0 samples.
B: Heavy and light fractions from all three replicates of ^{12}C-acetate treatments.
C: Heavy and light fractions from all three replicates of ^{13}C-acetate treatments.

Figure S6

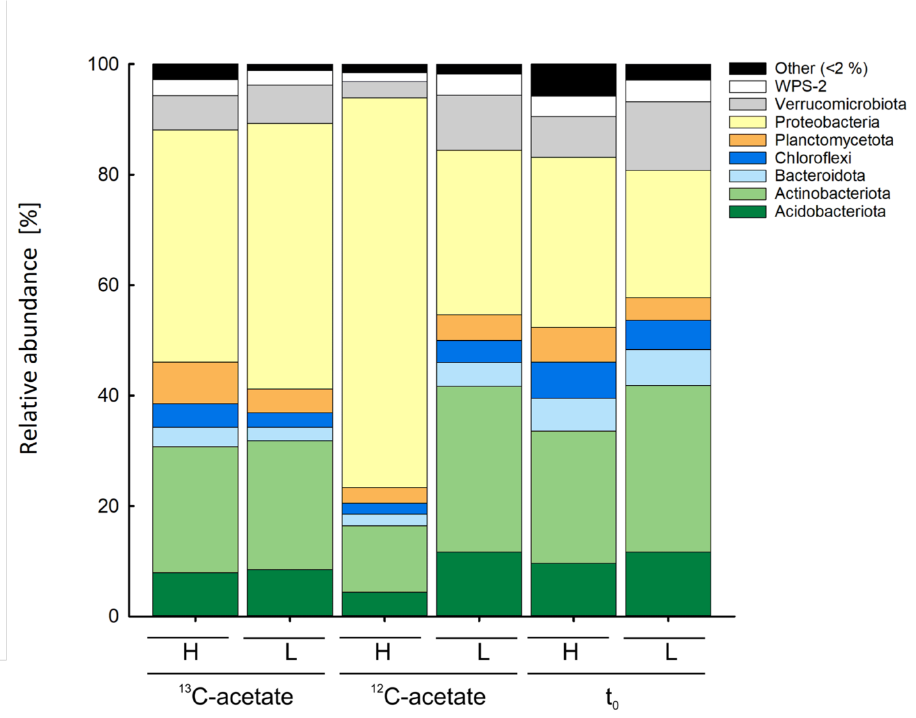

Figure S6. Mean relative abundance of bacterial phyla (>2% of relative abundance in at least one sample) delineated from analysis of density resolved 16S rRNA retrieved from cryoturbated peat circle sediments prior to and after 16 days of anoxic incubation (see Figure 1). Values are means of triplicate incubations. Heavy (H) and Light (L) indicate fractions representing ^{13}C-labeled and unlabeled 16S rRNA after isopycnic centrifugation (see Materials and Methods for further details) where sequences have been retrieved from. ^{13}C- and ^{12}C-acetate refer to treatments with ^{13}C- and ^{12}C-acetate, respectively. t_0 indicates peat circle sediments prior to incubation.

Figure S7

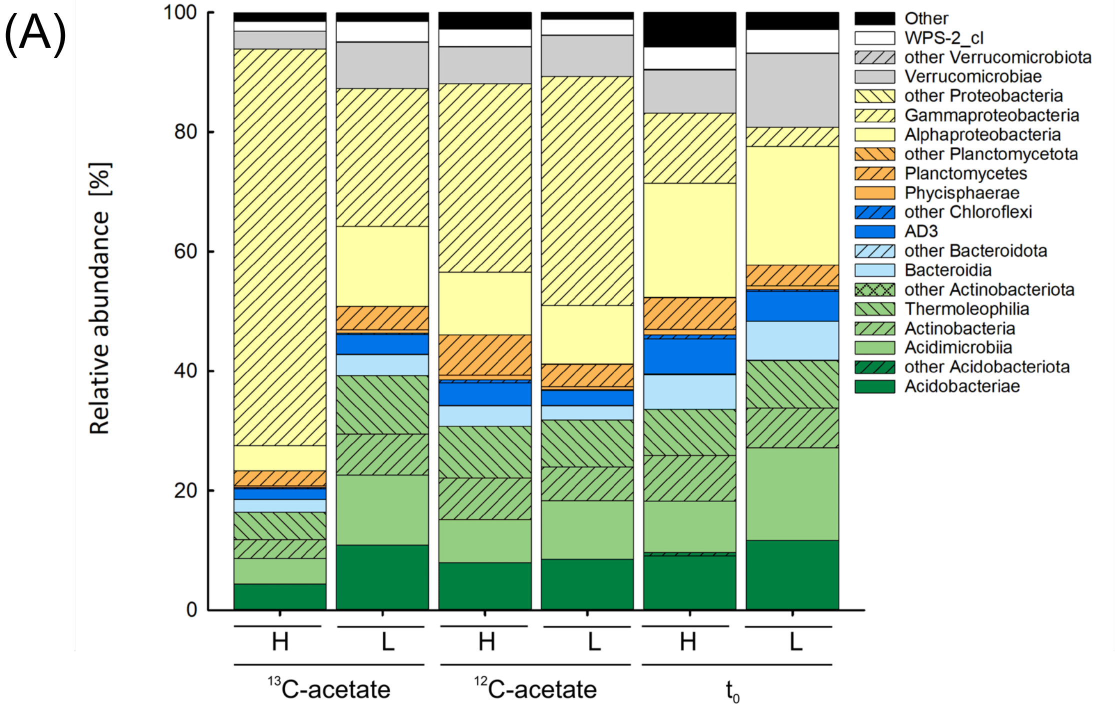

(A)
Relative abundance [%]
0
20
40
60
80
100
H
L
H
L
H
L
13C-acetate
12C-acetate
t0
Other
WPS-2_cl
other Verrucomicrobiota
Verrucomicrobiae
other Proteobacteria
Gammaproteobacteria
Alphaproteobacteria
other Planctomycetota
Planctomycetes
Phycisphaerae
other Chloroflexi
AD3
other Bacteroidota
Bacteroidia
other Actinobacteriota
Thermoleophilia
Actinobacteria
Acidimicrobiia
other Acidobacteriota
Acidobacteriae

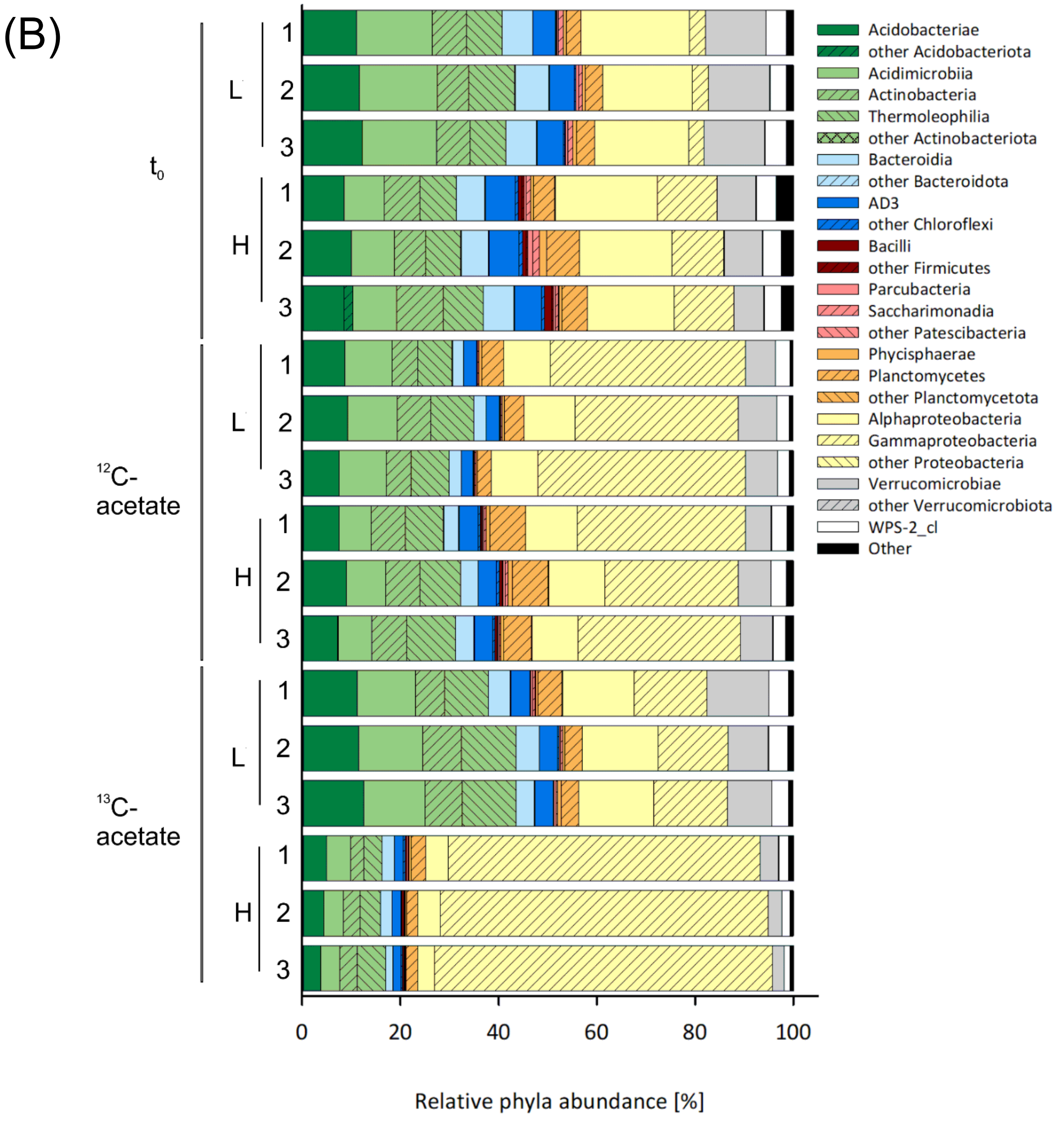

(B)
t0
12C-acetate
13C-acetate
L
H
1
2
3
0
20
40
60
80
100
Relative phyla abundance [%]
Acidobacteriae
other Acidobacteriota
Acidimicrobiia
Actinobacteria
Thermoleophilia
other Actinobacteriota
Bacteroidia
other Bacteroidota
AD3
other Chloroflexi
Bacilli
other Firmicutes
Parcubacteria
Saccharimonadia
other Patescibacteria
Phycisphaerae
Planctomycetes
other Planctomycetota
Alphaproteobacteria
Gammaproteobacteria
other Proteobacteria
Verrucomicrobiae
other Verrucomicrobiota
WPS-2_cl
Other

Figure S7. Mean relative abundance of bacterial classes (>2% of relative abundance in at least one sample) delineated from analysis of density resolved 16S rRNA retrieved from cryoturbated peat circle sediments prior to and after 16 days of anoxic incubation (see Figure 1). Values are means of triplicate incubations (A) or indicate single replicates (B). Heavy (H) and Light (L) indicate fractions representing ^{13}C-labeled and unlabeled 16S rRNA after isopycnic centrifugation (see Materials and Methods for further details) where sequences have been retrieved from. ^{13}C- and ^{12}C-acetate refer to treatments with ^{13}C- and ^{12}C-acetate, respectively. t_0 indicates peat circle sediments prior to incubation.

Figure S8

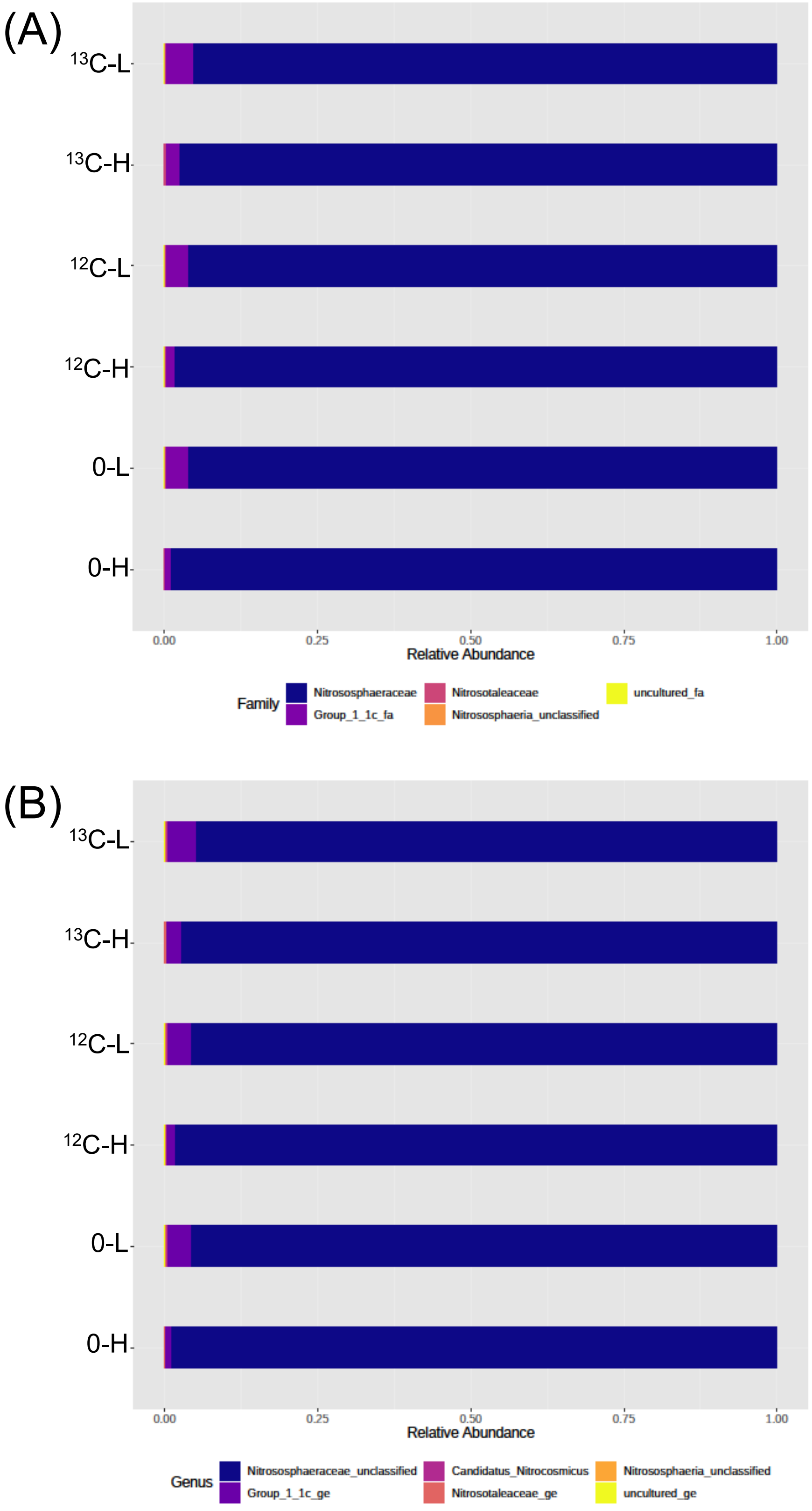

Figure S8. Mean relative abundance of archaeal families (A) and genera (B) delineated from analysis of density resolved 16S rRNA retrieved from cryoturbated peat circle sediments prior to and after 16 days of anoxic incubation (see Figure 1). Values are means of triplicate incubations. Sample code: H and L indicate heavy and light fractions, respectively; 0, ^{13}C- and ^{12}C- represent peat circle sediments prior to incubation, ^{13}C- and ^{12}C-acetate treatments, respectively.

Figure S9

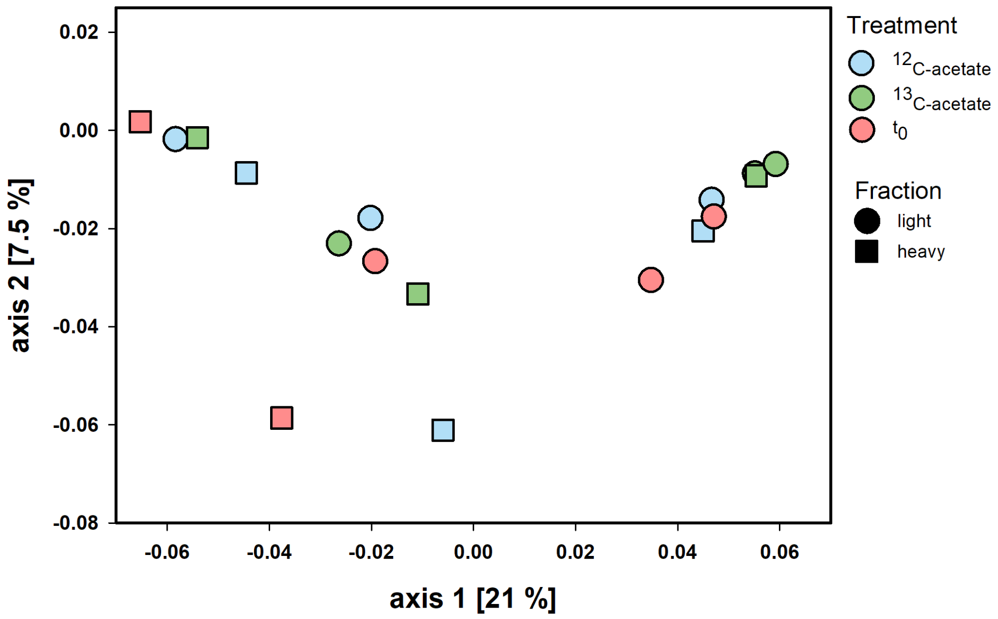

Figure S9. Principal Coordinates Analysis (PCoA) based on Bray-Curtis dissimilarity calculated from relative abundance data of archaeal species-level OTUs from density resolved 16S rRNA retrieved from cryoturbated peat circle sediments prior to (t_0) and after 16 days of anoxic incubation (see Figure 1).

Table S1. Correlation table of network analysis on bacterial genus level using SparCC algorithm (Figure 5A). Significant correlations of *Burkholderia-Caballeronia-Paraburkholderia* and *Rhodanobacter* spp. affiliating sequences are shown.

**Genus-level Taxon 1**	**Genus-level Taxon 2**	**Correlation**	**p-value**
Burkholderia_Caballeronia_Paraburkholderia	37_13_ge	-0.5083	0.0297
Burkholderia_Caballeronia_Paraburkholderia	*Acetobacteraceae*_unclassified	0.8124	0.0099
Burkholderia_Caballeronia_Paraburkholderia	*Acidipila*	0.4913	0.0495
Burkholderia_Caballeronia_Paraburkholderia	*Acidobacteriaceae*_(Subgroup_1)_unclassified	0.5336	0.0396
Burkholderia_Caballeronia_Paraburkholderia	*Acidocella*	0.5518	0.0198
Burkholderia_Caballeronia_Paraburkholderia	*Acidothermus*	0.7593	0.0099
Burkholderia_Caballeronia_Paraburkholderia	*Aquisphaera*	0.9022	0.0099
Burkholderia_Caballeronia_Paraburkholderia	*Asticcacaulis*	0.5135	0.0396
Burkholderia_Caballeronia_Paraburkholderia	B12_WMSP1_ge	0.7295	0.0099
Burkholderia_Caballeronia_Paraburkholderia	*Bacteroidota_unclassified*	0.6141	0.0099
Burkholderia_Caballeronia_Paraburkholderia	*Burkholderiaceae_unclassified*	0.5359	0.0198
Burkholderia_Caballeronia_Paraburkholderia	*Candidatus_Nostocoida*	0.9138	0.0099
Burkholderia_Caballeronia_Paraburkholderia	*Candidatus_Ovatusbacter*	-0.5198	0.0099
Burkholderia_Caballeronia_Paraburkholderia	*Conexibacter*	0.8632	0.0099
Burkholderia_Caballeronia_Paraburkholderia	*Frankiales_unclassified*	0.7387	0.0297
Burkholderia_Caballeronia_Paraburkholderia	*Gemmata*	0.5819	0.0198
Burkholderia_Caballeronia_Paraburkholderia	*Gemmatimonadaceae_unclassified*	0.6845	0.0099
Burkholderia_Caballeronia_Paraburkholderia	*Gemmatimonas*	0.4979	0.0495
Burkholderia_Caballeronia_Paraburkholderia	*Granulicella*	0.6775	0.0198
Burkholderia_Caballeronia_Paraburkholderia	IMCC26256_ge	0.5904	0.0198
Burkholderia_Caballeronia_Paraburkholderia	*Isosphaeraceae_unclassified*	0.9104	0.0099
Burkholderia_Caballeronia_Paraburkholderia	*Jatrophihabitans*	0.7261	0.0099
Burkholderia_Caballeronia_Paraburkholderia	*Legionella*	-0.6195	0.0297
Burkholderia_Caballeronia_Paraburkholderia	*Mucilaginibacter*	0.5412	0.0297
Burkholderia_Caballeronia_Paraburkholderia	*Novosphingobium*	0.5869	0.0396
Burkholderia_Caballeronia_Paraburkholderia	*Occallatibacter*	0.7075	0.0099
Burkholderia_Caballeronia_Paraburkholderia	*Pajaroellobacter*	-0.5466	0.0396
Burkholderia_Caballeronia_Paraburkholderia	*Pedosphaeraceae_ge*	0.6921	0.0099
Burkholderia_Caballeronia_Paraburkholderia	*Phenylobacterium*	0.6286	0.0396
Burkholderia_Caballeronia_Paraburkholderia	RCP2_54_ge	0.7328	0.0198
Burkholderia_Caballeronia_Paraburkholderia	*Rhodanobacter*	0.6159	0.0099
Burkholderia_Caballeronia_Paraburkholderia	*Rickettsiaceae_unclassified*	-0.5811	0.0297
Burkholderia_Caballeronia_Paraburkholderia	*Solirubrobacteraceae_unclassified*	0.6492	0.0297
Burkholderia_Caballeronia_Paraburkholderia	*Solirubrobacterales_unclassified*	0.487	0.0396
Burkholderia_Caballeronia_Paraburkholderia	Subgroup_13_ge	0.5191	0.0198
Burkholderia_Caballeronia_Paraburkholderia	Subgroup_2_ge	0.8369	0.0198
Burkholderia_Caballeronia_Paraburkholderia	*Tepidisphaerales_unclassified*	0.7028	0.0099
Burkholderia_Caballeronia_Paraburkholderia	*Tundrisphaera*	0.8681	0.0099
Burkholderia_Caballeronia_Paraburkholderia	WD2101_soil_group_ge	0.5906	0.0396
Burkholderia_Caballeronia_Paraburkholderia	WPS_2_ge	0.6062	0.0099

Table S1 (continued). Correlation table of network analysis on bacterial genus level using SparCC algorithm (see Figure 5A). Significant correlations of *Burkholderia-Caballeronia-Paraburkholderia* and *Rhodanobacter* spp. affiliating sequences are shown.

Genus-level Taxon 1	Genus-level Taxon 2	Correlation	p-value
Rhodanobacter	67_14_ge	0.7189	0.0099
Rhodanobacter	*Acetobacteraceae*_unclassified	0.6952	0.0099
Rhodanobacter	*Acidicaldus*	0.6046	0.0297
Rhodanobacter	*Acidimicrobiia*_unclassified	0.8753	0.0099
Rhodanobacter	*Acidiphilium*	0.565	0.0396
Rhodanobacter	*Acidipila*	0.8737	0.0099
Rhodanobacter	*Acidobacteriaceae*_(Subgroup_1)_unclassified	0.8053	0.0099
Rhodanobacter	*Acidothermus*	0.8716	0.0099
Rhodanobacter	AD3_ge	0.8077	0.0099
Rhodanobacter	*Anaerococcus*	-0.5117	0.0297
Rhodanobacter	*Aquisphaera*	0.715	0.0198
Rhodanobacter	*Asticcacaulis*	0.6313	0.0198
Rhodanobacter	B10_SB3A_ge	-0.7032	0.0099
Rhodanobacter	*Bacteria*_unclassified	0.6577	0.0495
Rhodanobacter	*Bacteroidota*_unclassified	0.6721	0.0297
Rhodanobacter	*Bauldia*	0.6846	0.0198
Rhodanobacter	*Beijerinckiaceae*_unclassified	0.6147	0.0198
Rhodanobacter	*Bryobacter*	0.6329	0.0198
Rhodanobacter	*Burkholderia_Caballeronia_Paraburkholderia*	0.6159	0.0099
Rhodanobacter	*Burkholderiaceae*_unclassified	0.7673	0.0099
Rhodanobacter	*Candidatus_Solibacter*	0.5734	0.0099
Rhodanobacter	*Caulobacteraceae*_unclassified	0.7944	0.0099
Rhodanobacter	*Chloroflexi*_unclassified	-0.4809	0.0495
Rhodanobacter	*Conexibacter*	0.8495	0.0297
Rhodanobacter	*Cutibacterium*	-0.6078	0.0099
Rhodanobacter	*Devosiaceae*_unclassified	0.8543	0.0099
Rhodanobacter	*Edaphobacter*	0.801	0.0099
Rhodanobacter	*Enterobacteriaceae*_unclassified	-0.6285	0.0198
Rhodanobacter	*Frankiales*_unclassified	0.8164	0.0099
Rhodanobacter	*Gaiellales*_unclassified	0.7761	0.0099
Rhodanobacter	*Galbitalea*	0.8499	0.0099
Rhodanobacter	*Gemmataceae*_unclassified	0.4778	0.0396
Rhodanobacter	*Granulicella*	0.9096	0.0099
Rhodanobacter	*Iamia*	0.8828	0.0099
Rhodanobacter	IMCC26256_ge	0.8993	0.0099
Rhodanobacter	*Isosphaeraceae*_unclassified	0.6114	0.0198
Rhodanobacter	*Janthinobacterium*	-0.7784	0.0099
Rhodanobacter	*Jatrophihabitans*	0.7414	0.0297
Rhodanobacter	KF_JG30_B3_ge	0.7278	0.0198
Rhodanobacter	*Lawsonella*	-0.56	0.0198
Rhodanobacter	*Leuconostoc*	-0.4848	0.0297
Rhodanobacter	*Micropepsaceae*_unclassified	0.8316	0.0099
Rhodanobacter	*Mucilaginibacter*	0.784	0.0099
Rhodanobacter	*Occallatibacter*	0.9115	0.0099
Rhodanobacter	*Paracoccus*	-0.5079	0.0495
Rhodanobacter	*Pedosphaeraceae_ge*	0.8516	0.0099
Rhodanobacter	*Phenylobacterium*	0.7777	0.0297
Rhodanobacter	*Pseudolabrys*	0.6153	0.0099
Rhodanobacter	*Pseudomonas*	-0.5055	0.0396
Rhodanobacter	RCP2_54_ge	0.8097	0.0099
Rhodanobacter	*Roseiarcus*	0.7133	0.0198
Rhodanobacter	*Saccharimonadales*_unclassified	0.455	0.0396
Rhodanobacter	*Solirubrobacteraceae*_unclassified	0.8568	0.0198
Rhodanobacter	*Solirubrobacterales*_unclassified	0.8588	0.0099
Rhodanobacter	*Sphingomonadaceae*_unclassified	0.4842	0.0297
Rhodanobacter	*Staphylococcus*	-0.5193	0.0198
Rhodanobacter	Subgroup_13_ge	0.5406	0.0396
Rhodanobacter	Subgroup_2_ge	0.8628	0.0495
Rhodanobacter	*Tepidisphaerales*_unclassified	0.6729	0.0297
Rhodanobacter	*Thermoleophilia*_unclassified	0.7003	0.0099
Rhodanobacter	*Tundrisphaera*	0.6042	0.0099
Rhodanobacter	uncultured	0.8411	0.0099
Rhodanobacter	uncultured_ge	0.8642	0.0099
Rhodanobacter	*Verrucomicrobiaceae*_unclassified	-0.5151	0.0297
Rhodanobacter	WD2101_soil_group_ge	0.6635	0.0297
Rhodanobacter	WD260_ge	0.6857	0.0099
Rhodanobacter	WPS_2_ge	0.8374	0.0099
Rhodanobacter	*Xanthobacteraceae*_unclassified	0.5919	0.0495
